# Peroxisome Proliferator Activated Receptor-α/Hypoxia Inducible Factor-1α Interplay Sustains Carbonic Anhydrase IX and Apoliprotein E Expression in Breast Cancer Stem Cells

**DOI:** 10.1371/journal.pone.0054968

**Published:** 2013-01-25

**Authors:** Alessio Papi, Gianluca Storci, Tiziana Guarnieri, Sabrina De Carolis, Sara Bertoni, Nicola Avenia, Alessandro Sanguinetti, Angelo Sidoni, Donatella Santini, Claudio Ceccarelli, Mario Taffurelli, Marina Orlandi, Massimiliano Bonafé

**Affiliations:** 1 Department of Biological, Geological, and Environmental Sciences, University of Bologna, Bologna, Italy; 2 Department of Experimental, Diagnostic, and Specialty Medicine, University of Bologna, Bologna, Italy; 3 Center for Applied Biomedical Research (CRBA), St. Orsola-Malpighi University Hospital, Bologna, Italy; 4 National Biostructures and Biosystems Institute (INBB), Rome, Italy; 5 Department of Surgical Sciences, Radiology and Dentistry, University of Perugia, Perugia, Italy; 6 Department of Experimental Medicine and Biochemical Sciences, University of Perugia, Perugia, Italy; 7 Department of Radiology and Histo-cytopathology, St. Orsola-Malpighi University Hospital, Bologna, Italy; 8 Department of Clinical and Surgical Sciences, University of Bologna, Bologna, Italy; University Medical Center Freiburg, Germany

## Abstract

**Aims:**

Cancer stem cell biology is tightly connected to the regulation of the pro-inflammatory cytokine network. The concept of cancer stem cells “inflammatory addiction” leads to envisage the potential role of anti-inflammatory molecules as new anti-cancer targets. Here we report on the relationship between nuclear receptors activity and the modulation of the pro-inflammatory phenotype in breast cancer stem cells.

**Methods:**

Breast cancer stem cells were expanded as mammospheres from normal and tumor human breast tissues and from tumorigenic (MCF7) and non tumorigenic (MCF10) human breast cell lines. Mammospheres were exposed to the supernatant of breast tumor and normal mammary gland tissue fibroblasts.

**Results:**

In mammospheres exposed to the breast tumor fibroblasts supernatant, autocrine tumor necrosis factor-α signalling engenders the functional interplay between peroxisome proliferator activated receptor-α and hypoxia inducible factor-1α (PPARα/HIF1α). The two proteins promote mammospheres formation and enhance each other expression via miRNA130b/miRNA17-5p-dependent mechanism which is antagonized by PPARγ. Further, the PPARα/HIF1α interplay regulates the expression of the pro-inflammatory cytokine interleukin-6, the hypoxia survival factor carbonic anhydrase IX and the plasma lipid carrier apolipoprotein E.

**Conclusion:**

Our data demonstrate the importance of exploring the role of nuclear receptors (PPARα/PPARγ) in the regulation of pro-inflammatory pathways, with the aim to thwart breast cancer stem cells functioning.

## Introduction

Breast cancer is a heterogeneous set of diseases that constitute the leading cause of cancer among women in western countries [1], [2]. In the recent past, a minor sub-population of tumor cells endued with the characteristics of stem cells (named cancer stem cells, CSCs) has been identified [3]–[5]. It is currently proposed that CSCs provide the cellular substrate for metastatic spreading and relapse and constitute the ultimate targets for innovating cancer therapy [4]–[6]. CSCs can be studied *in vitro* by expanding multicellular spheroids (mammospheres, MS) from breast cancer surgical specimens and cell lines [7]–[9]. The pro-inflammatory cytokine network is of key importance in breast CSCs biology [9], [10]. In particular, the pro-inflammatory nuclear factor-κB (NF-κB) pathway, as well the NF-κB-regulated cytokines tumor necrosis factor α (TNFα) and interleukin 6 (IL6) trigger MS survival and self-renewal [9]–[13]. In haematopoietic and prostate CSCs, such a pro-inflammatory phenotype has been associated with a kind of “inflammatory addiction”, which makes CSCs likely targets of anti-inflammatory drugs, that may act as potential enhancers of cancer therapy [14]–[16].

The stromal cell is the cornerstone of the stem cell niche [17], [18]. Stroma-derived inflammatory mediators, such as prostaglandins and IL6 promote MS growth and survival [9], [19]. Similarly to its normal counterpart [20], the CSCs niche is characterized by low oxygen tension (hypoxia) which promotes stem cell survival [21]. Hypoxia inducible factor1α (HIF1α) affects a variety of malignant features, such as hypoxic cancer cell survival, via the regulation of a large number of genes, including carbonic anhydrase IX (CAIX) [22], [23].

We recently reported that peroxisome proliferator activated receptor-α (PPARα) modulates the expression of stem cell genes (e.g. Jagged1) and apolipoprotein E (ApoE) in breast CSCs [24]; ApoE is a lipoprotein over-expressed in MS [7]. PPARα belongs to the PPAR nuclear receptor family, enlisting also PPARβ and PPARγ among its members. PPARα plays a key role in lipid metabolism and it is activated by fatty acids, leukotriene and synthetic fibrates [25]. PPARγ binds natural molecules, such as prostaglandin J2, polyunsaturated fatty acids, and synthetic compounds, such as Pioglitazone (PGZ) [26]. Ligands of PPARγ reduce the viability of cancer cell lines and breast CSCs [24], [27]–[29]. Noteworthy, PPARγ plays a significant inhibitory role on the inflammatory process [30]–[31], while PPARα exerts pro-inflammatory activity [32]. Interestingly, the expression of PPARα increases, while that of PPARγ decreases in neural stem cells exposed to hypoxia [33]. In this study, MS from normal (N-MS) and tumor (T-MS) tissues, as well as from tumorigenic MCF7 (MCF7-MS) and non tumorigenic (MCF10-MS) human breast cell lines, were exposed to the supernatant of normal mammary gland and breast tumor associated fibroblasts. We undertook this approach to elucidate the regulation of PPARα and PPARγ in the context of breast CSCs inflammatory pathway activation.

## Results

### Enhanced Autocrine TNFα Loop in Breast Cancer Tissue Derived MS Exposed to the Supernatant of Tumor Associated Fibroblasts

**Figure 1:**
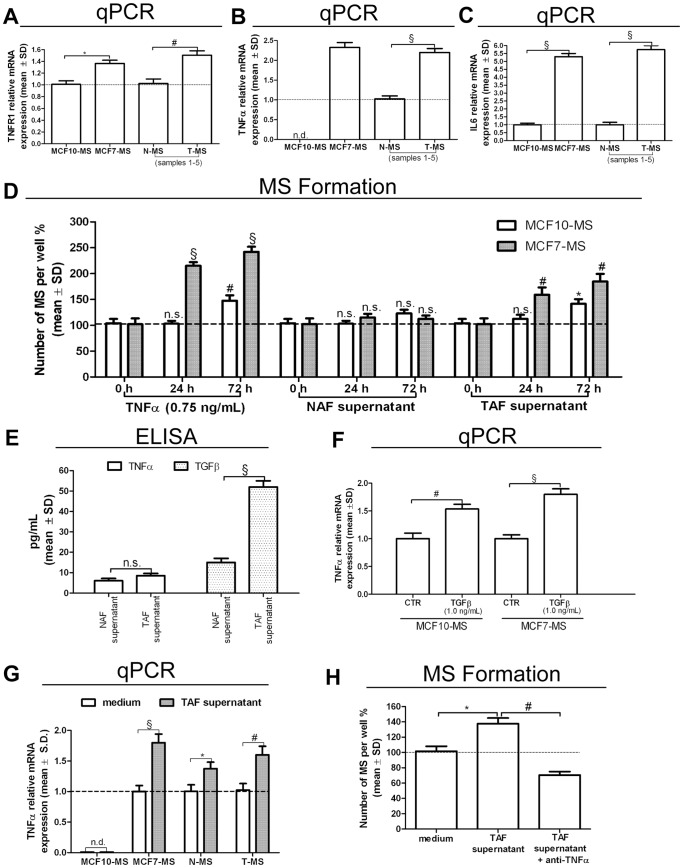
Autocrine TNFα loop in MS exposed to the TAF supernatant. TNFR1 (A), TNFα (B) and IL6 (C) real-time reverse transcriptase quantitative (qPCR) mRNA analysis in MCF7-MS, MCF10-MS, T-MS and N-MS (samples 1–5). (D) MS formation assay in TNFα (0.75 ng/mL), NAF and TAF supernatant (10% final concentration)-exposed MCF10 and MCF7 for 24 to 72 h; (E) TNFα and TGFβ ELISA test on TAF and NAF supernatants (samples 6–12); (F) TNFα mRNA qPCR analysis in TGFβ (1.0 ng/mL, 24 h)-exposed MCF10-MS and MCF7-MS (G) TNFα mRNA qPCR analysis in TAF supernatant (10%, 24 h)-exposed MCF10/MCF7-MS, and in N−/T-MS (samples 5–6, n = 2); (H) TAF supernatant-induced T-MS formation assay in presence/absence of TNFα inhibitory antibody (1.5 µg/mL, 24 h, sample 13). Data are expressed as mean ± Standard Deviation (S.D.), n = 3 unless otherwise specified, *p<0.05, #*p*<0.01, §*p*<0.005, ANOVA test. n.s.: not significant.

We recently reported the increase of NF-κB activity in breast tumor MS (T-MS), compared to their normal counterparts (N-MS) [24]. The major trigger of NF-κB pathway is TNFα, a potent inducer of MS formation [12], [13]. Here we found higher expression of tumor necrosis factor receptor-1 (TNFR1), and of the NF-κB targets TNFα and IL6 in tumorigenic MCF7-MS and T-MS, compared to non tumorigenic MCF10-MS and N-MS, respectively (Figure 1A, B, C). We also observed that exogenous TNFα elicited MS formation in MCF7 to a higher extent than in MCF10 cells, a phenomenon that was mimicked by the administration of the supernatant of tumor associated fibroblasts (TAF), but not of normal mammary gland fibroblasts (NAF, Figure 1D). Both TAF and NAF secreted very low levels of TNFα, whereas TAF secreted higher levels of transforming growth factor-β1 (TGFβ) compared to NAF (Figure 1E). TGFβ is a potent MS growth factor and synergizes with TNFα to induce stem cell features in breast cancer cells [34]–[35]. With respect to this issue, we observed that TGFβ was able to induce TNFα expression in MS (Figure 1F). Moreover, the TAF supernatant induced TNFα expression in T-MS and MCF7-MS to a higher extent than in N-MS and MCF10-MS, respectively (Figure 1G). Finally, the increase in T-MS formation following the TAF supernatant administration was halted by TNFα blocking antibody administration (Figure 1H). These data show that autocrine TNFα signalling in breast CSCs is enhanced by TAF secretion of TGFβ.

### The Tumor Associated Fibroblasts Supernatant Elicits the PPARα/HIF1α Interplay Dependent Growth of MS

**Figure 2:**
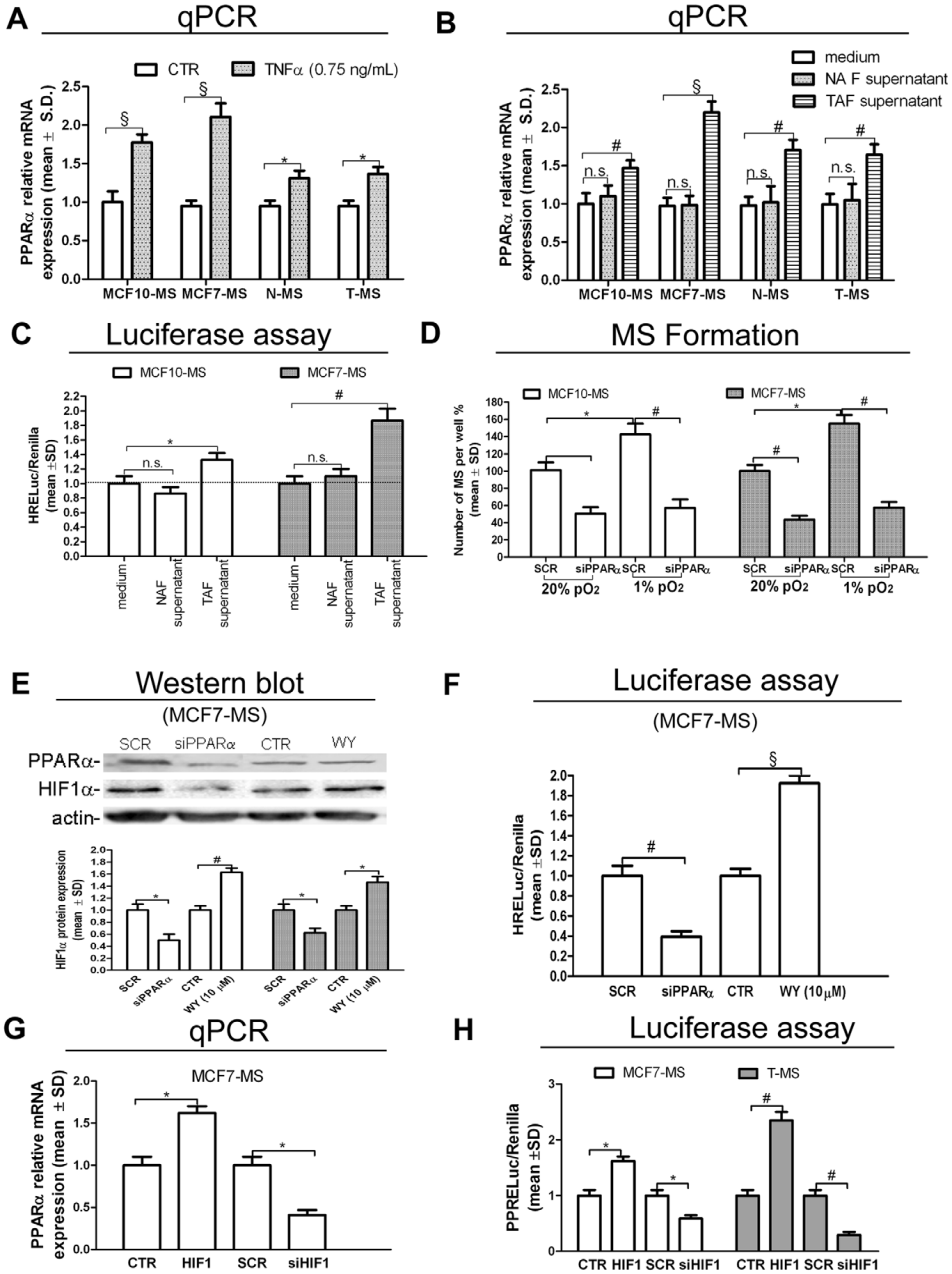
The TAF supernatant induces the PPARα/HIF1α interplay and promotes T-MS growth. PPARα mRNA qPCR analysis in MCF10/MCF7-MS and N−/T-MS (samples 14 and 15) upon exposure to: (A) TNFα (0.75 ng/mL, 24 h), (B) NAF or TAF supernatant (10%, 24 h). (C) HRELuc activity in NAF or TAF supernatant (10%, 24 h)-exposed MCF10/MCF7-MS. (D) MS formation assay in scramble (SCR)/siPPARα (72 h)-transfected MCF10 and MCF7 cells in normoxia (20% pO_2_) or hypoxia (1% pO_2_). HIF1α and PPARα protein expression (E) and HRELuc activity (F) in SCR/siPPARα (72 h)-transfected or PPARα agonist WY (10 µM, 24 h)-exposed hypoxic MCF7-MS. PPARα mRNA qPCR analysis (G) and PPRELuc activity (H) in HIF1 vector (48 h) and SCR/siHIF1 (72 h)-transfected MCF7-MS and T-MS (samples 15–17). Data are expressed as mean ±S.D., n = 3 unless otherwise specified, *p<0.05, ^#^ *p*<0.01, ^§^ *p*<0.005, ANOVA test. n.s.: not significant.

We recently reported that PPARα promotes tumor MS formation and the expression of the MS growth factor Jagged1 [24]. In MS exposed to TNFα, we observed increased expression of PPARα (Figure 2A) and Jagged1 (+153%, *p*<0.005, in MCF10-MS; +167%, *p*<0.005, in MCF7-MS; +63%, *p*<0.05, in N-MS; +76%, *p*<0.05 in T-MS, Figure S1A). Moreover, in the same cellular models, the administration of the TAF supernatant up-regulated PPARα (Figure 2B and +54%, *p*<0.05, in MCF7-MS, Figure S1B) and Jagged1 (+283%, *p*<0.005, in MCF10-MS; +297%, *p*<0.005, in MCF7-MS; +253%, *p*<0.05, in N-MS; +266%, *p*<0.05 in T-MS, Figure S1C) to a higher extent than the NAF supernatant. We previously demonstrated that HIF1α is a TNFα target [12]. Moreover, literature data report that HIF1α is a potential PPARα target [33]. Here, we observed that exposure of MCF7-MS and MCF10-MS to the TAF supernatant elicited HIF1α transcriptional activity (Figure 2C) and mRNA expression (+65%, *p*<0.05, in MCF10-MS; +76%, *p*<0.01, in MCF7-MS, Figure S2A). Exposure to hypoxia, meanwhile inducing HIF1α expression and activity (+63%, *p*<0.05, in MCF10-MS; +85%, *p*<0.01, in MCF7-MS, Figure S2B; +94%, *p*<0.005, in MCF10-MS; +122%, *p*<0.005, in MCF7-MS, Figure S2C), was responsible for an increase in PPARα expression and MS formation (Figure 2D). The phenomenon was hampered by siRNA-mediated PPARα knock-down (KD) in normoxic and hypoxic conditions (−63%, *p*<0.005 and −56%, *p*<0.005 respectively, Figure S2D). Prompted by these data, we investigated the relationship between PPARα and HIF1α expression. We observed that siRNA-PPARα administration reduced, while the PPARα agonist Wy16463 (WY) triggered the expression of HIF1α protein expression and transcriptional activity (Figure 2E and Figure 2F). HIF1α expression was increased also in MCF10-MS exposed to WY (+91%, *p*<0.01, Figure S2E). As a further insight into the PPARα/HIF1α interplay, we found that PPARα expression and peroxisome proliferator response element reporter (PPRELuc) activity were elicited by HIF1α over-expression and reduced by siRNA-HIF1 in MCF7-MS and T-MS (Figure 2G and Figure 2H). Finally, we verified that the over-expression of PPARα up-regulated two inducers of MS formation [9], [12], namely SLUG (+41%, *p*<0.05, in MCF10-MS, +62%, *p*<0.01 in MCF7-MS, Figure S3A) and IL6 (+182%, *p*<0.01, in MCF10-MS; +552%, *p*<0.005, in MCF7-MS, Figure S3B). In MCF7-MS, HIF1α over-expression triggered, as well as HIF1α KD hindered the expression of SLUG (+72%, *p*<0.01, −43%, *p*<0.01, respectively, Figure S3C) and IL6 (+295%, *p*<0.005, −36%, *p*<0.05, respectively, Figure S3D). These data suggest that the PPARα/HIF1α interplay is active in T-MS to a higher extent than in their normal counterpart and that it drives the expression of MS growth promoting genes.

### PPARγ Expression Antagonizes the PPARα/HIF1α Interplay

**Figure 3:**
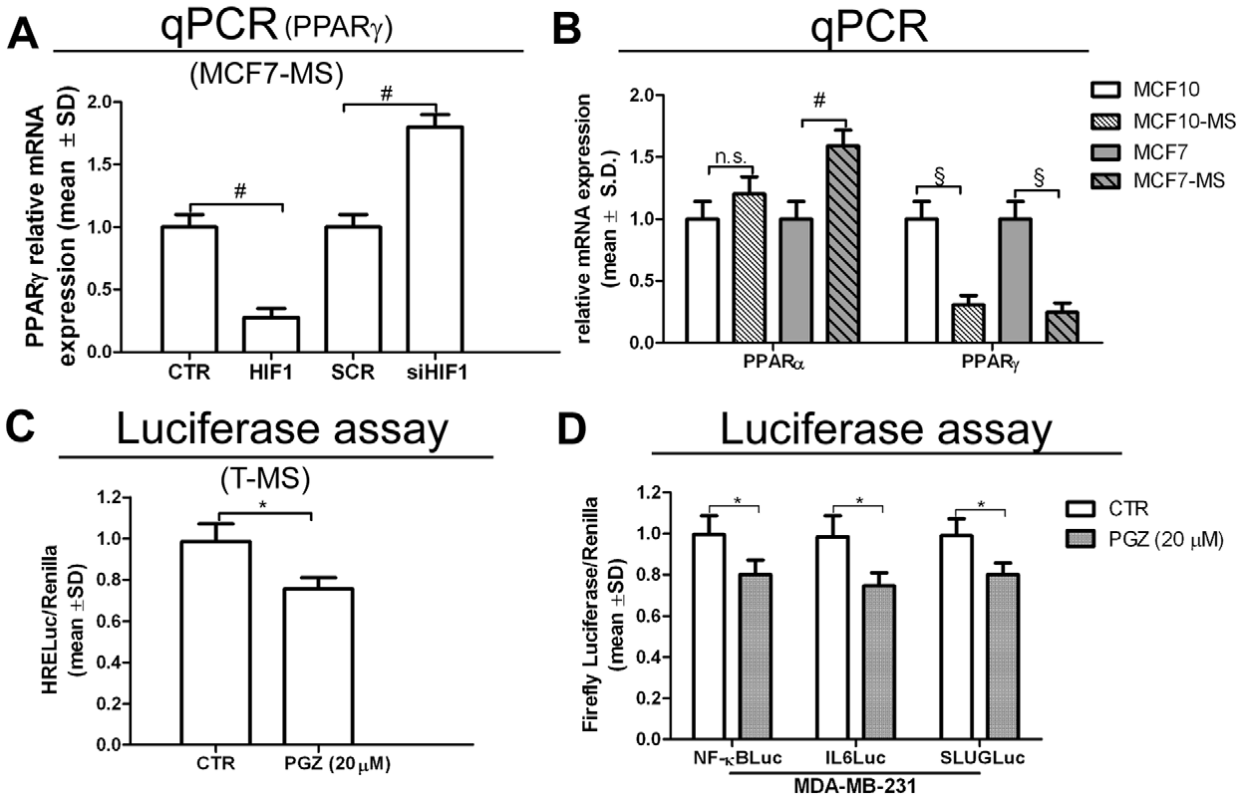
PPARγ antagonizes the PPARα/HIF1α interplay and inhibits pro-inflammatory CSCs pathways. (A) PPARγ mRNA qPCR analysis in HIF1 vector (48 h) or SCR/siHIF1 (72 h)-transfected MCF7-MS. (B) PPARα and PPARγ mRNA qPCR analysis in adherent MCF10 *vs* MCF10-MS and in adherent MCF7 *vs* MCF7-MS; (C) HRELuc activity in hypoxic T-MS (samples 15–16) exposed to PPARγ agonist PGZ (20 µM, 24 h) (D) NF-κBLuc, IL6Luc, SLUGLuc activity in MDA-MB-231 breast cancer cells exposed to PGZ (20 µM, 24 h). Data are expressed as mean ±S.D., n = 3, *p<0.05, ^#^ *p*<0.01, ^§^ *p*<0.005, ANOVA test. n.s.: not significant.

We pursued our investigation by observing that, opposite PPARα regulation (see Figure 2G), the transfection of HIF1 vector down-regulated (−59%, *p*<0.01, Figure S4A), as well as siHIF1 up-regulated PPARγ expression (Figure 3A). Interestingly, higher levels of PPARα mRNA and protein, but reduced mRNA PPARγ levels were found in MCF7-MS and MCF10-MS compared to adherent cells (Figure 3B). Accordingly, similar data were obtained by western blot analysis of PPARα (+42%, *p*<0.05, in MCF10-MS *vs* MCF10, and +82%, *p*<0.01, in MCF7-MS *vs* MCF7) and PPARγ (−22%, *p*<0.05, in MCF10-MS *vs* MCF10, and −10%, *p*>0.05, in MCF7-MS *vs* MCF7, Figure S4B). Owing to PPARγ needs to heterodimerize with retinoid X receptors (RXRs) to exert its transcriptional activity [28], we tested also RXRα, RXRβ and RXRγ expression in MS. As previously demonstrated in MCF7 cells [24], RXRα gene expression was reduced in MS compared to adherent MCF10 cells (−62%, *p*<0.01, Figure S4C). Moreover, siHIF1 transfection in MCF7-MS up-regulated RXRα (+83%, *p*<0.01) and PPARβ (+64%, *p*<0.01) expression (Figure S4D). The phenomenon paralleled the up-regulation of PPARγ expression in the same cells (see Figure 3A). We then observed that the PPARγ ligand PGZ reduced HRELuc activity in T-MS (Figure 3C) and HIF1α expression in MCF7-MS (−37%, *p*<0.05) and in T-MS (−26%, *p*<0.01, Figure S5A). Further, PGZ reduced MS formation (−23%, *p*<0.05, in MCF7-MS and −27%, *p*<0.01, in T-MS, Figure S5B) and the expression of breast CSCs regulatory pathway [9] in hypoxic MCF7-MS (−25%, *p*<0.05 for IL6, −22%, *p*<0.05 for Notch3, −21%, *p*<0.05 for Jagged1, Figure S5C) and T-MS (−55%, *p*<0.05 for IL6, −57%, *p*<0.05 for Notch3, −51%, *p*<0.05 for Jagged1, Figure S5C). Finally, luciferase reporter assay showed that PGZ inhibited the activity of NF-κB and of the NF-κB targets IL6 and SLUG promoters (Figure 3D). In line with these data, PGZ inhibited the expression of IL6 (−19%, *p*<0.05), Interleukin 8 (IL8, −31%, *p*<0.05), SLUG (−18%, *p*<0.05) and TNFα (−54%, *p*<0.01) in the breast cancer estrogen receptor-α (ERα) negative cell line MDA-MB-231 (Figure S6). These data point out the reciprocal antagonistic role of PPARγ and PPARα in breast CSCs, in which the former facilitates and the latter opposes the expression of breast CSCs regulatory pathway.

### Opposing Roles of miRNA130b and miRNA17-5p on the PPARα/HIF1α Interplay

**Figure 4:**
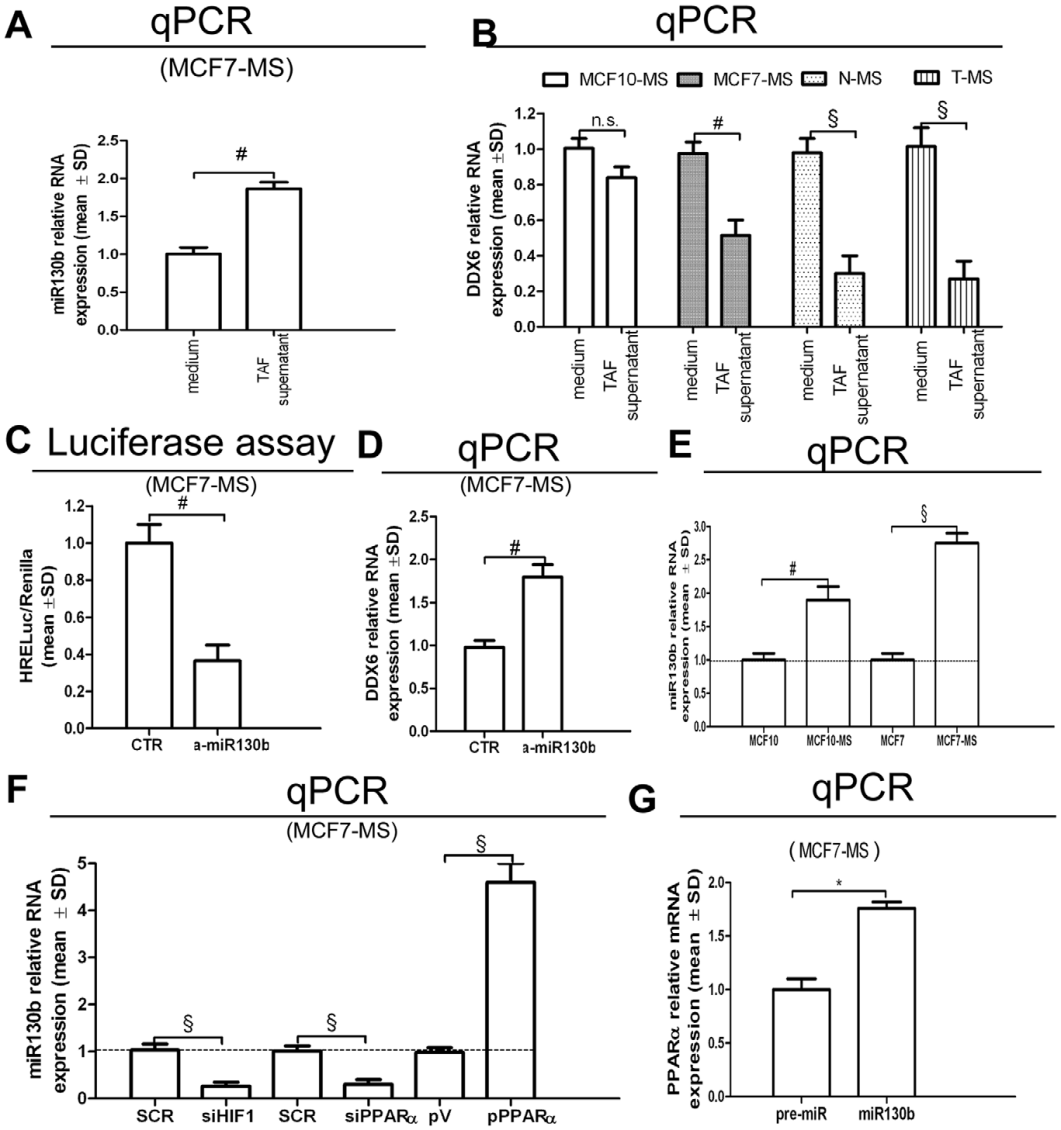
Role of miR130b on the PPARα/HIF1α interplay in breast CSCs. (A) miR130b qPCR analysis in TAF supernatant (10%, 24 h)-exposed MCF7-MS; (B) DDX6 qPCR analysis in TAF supernatant (10%, 24 h)-exposed MCF10/MCF7-MS and N−/T-MS (samples, 14 and 15); HRELuc assay (C) and DDX6 qPCR mRNA analysis (D) in AntagomiR130b (a-miR130b, 48 h)-transfected MCF7-MS; (E) miR130b qPCR analysis in MCF7 and MCF10 cultured as adherent or MS; (F) miR130b qPCR analysis in SCR/siHIF1/siPPARα (72 h) and pV/pPPARα vector (24 h)-transfected MCF7-MS; (G) PPARα qPCR analysis in pre-miR130b (48 h)-transfected MCF7-MS. Data are expressed as mean ±S.D., n = 3, *p<0.05, ^#^ *p*<0.01, ^§^ *p*<0.005, ANOVA test. n.s.: not significant.

To mechanistically elucidate the PPARα/HIF1α interplay, we examined microRNA130b (miR130b) expression. This microRNA was chosen basing on two considerations: i. its capability to increase HIF1α expression via the down-regulation of the HIF1α mRNA translation inhibitory protein DDX6 [36]; ii, the presence of its binding site at PPARγ mRNA 3′UTR [37]. We observed that the miR130b up-regulation in TAF supernatant-exposed MCF7-MS (Figure 4A) was paralleled by the reduction of DDX6 expression in MS (Figure 4B). Accordingly, the administration of miR130b antagonist (a-miR130b) in MCF7-MS reduced the activity of HRELuc (Figure 4C) and induced DDX6 expression (Figure 4D). We then observed the up-regulation of miR130b expression in MCF7- and MCF10-MS compared to adherent cells (Figure 4E). In keeping with the results above reported, miR130b expression was down-regulated by siHIF1 and siPPARα, and it was induced by PPARα over-expression in MCF7-MS (Figure 4F). Finally, the transfection of pre-miR130b increased PPARα expression in MCF7-MS (Figure 4G).

**Figure 5:**
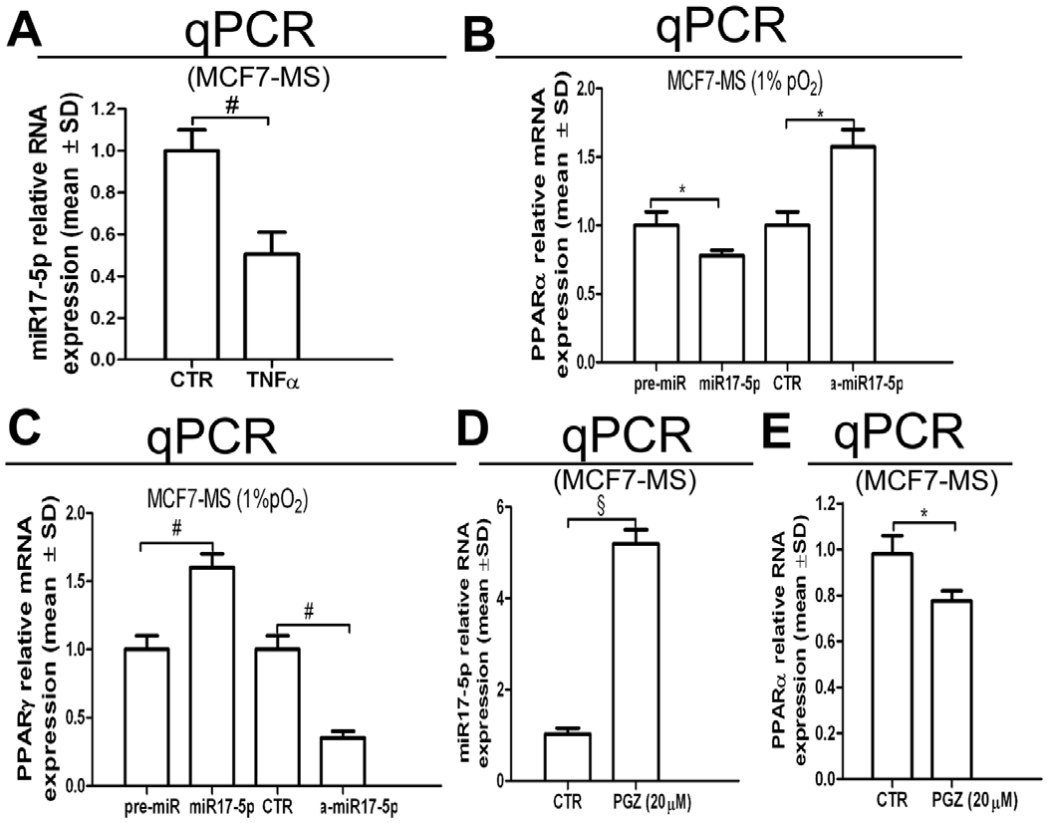
Role of miR17-5p on the PPARα/PPARγ interplay in breast CSCs. (A) miR17-5p qPCR analysis in TNFα (0.75 ng/mL, 24 h)-exposed MCF7-MS. PPARα mRNA (B) and PPARγ mRNA (C) qPCR analysis in pre-miR17-5p or antago-miR17-5p (a-miR17-5p)-transfected MCF7-MS (48 h). miR17-5p (D) and PPARα mRNA (E) qPCR analysis in PGZ (20 µM, 24 h)-exposed MCF7-MS. Data are expressed as mean ±S.D., n = 3, *p<0.05, ^#^ *p*<0.01, ^§^ *p*<0.005, ANOVA test. n.s.: not significant.

Further insight into the PPARα/HIF1α interplay was obtained via the assessment of microRNA17-5p (miR17-5p), whose binding consensus is present at PPARα mRNA 3′UTR. In respect to this issue, we were allowed to observe that miR17-5p expression was decreased by TNFα administration in MCF7-MS (Figure 5A). Further, miR17-5p transfection down-regulated PPARα, as well as miR17-5p antagonist (a-miR17-5p) induced PPARα expression (Figure 5B). In the same experimental setting, miR17-5p increased PPARγ expression and a-miR17-5p reduced PPARγ expression (Figure 5C). Finally, the administration of PGZ increased miR17-5p expression (Figure 5D), while it dampened PPARα expression in MCF7-MS (Figure 5D). These data point out that the antagonist interplay between PPARα and PPARγ is mediated by miR130b and miR17-5p.

### CAIX Over-expression in Tumor MS is Under the Control of the PPARα/HIF1α Interplay

**Figure 6:**
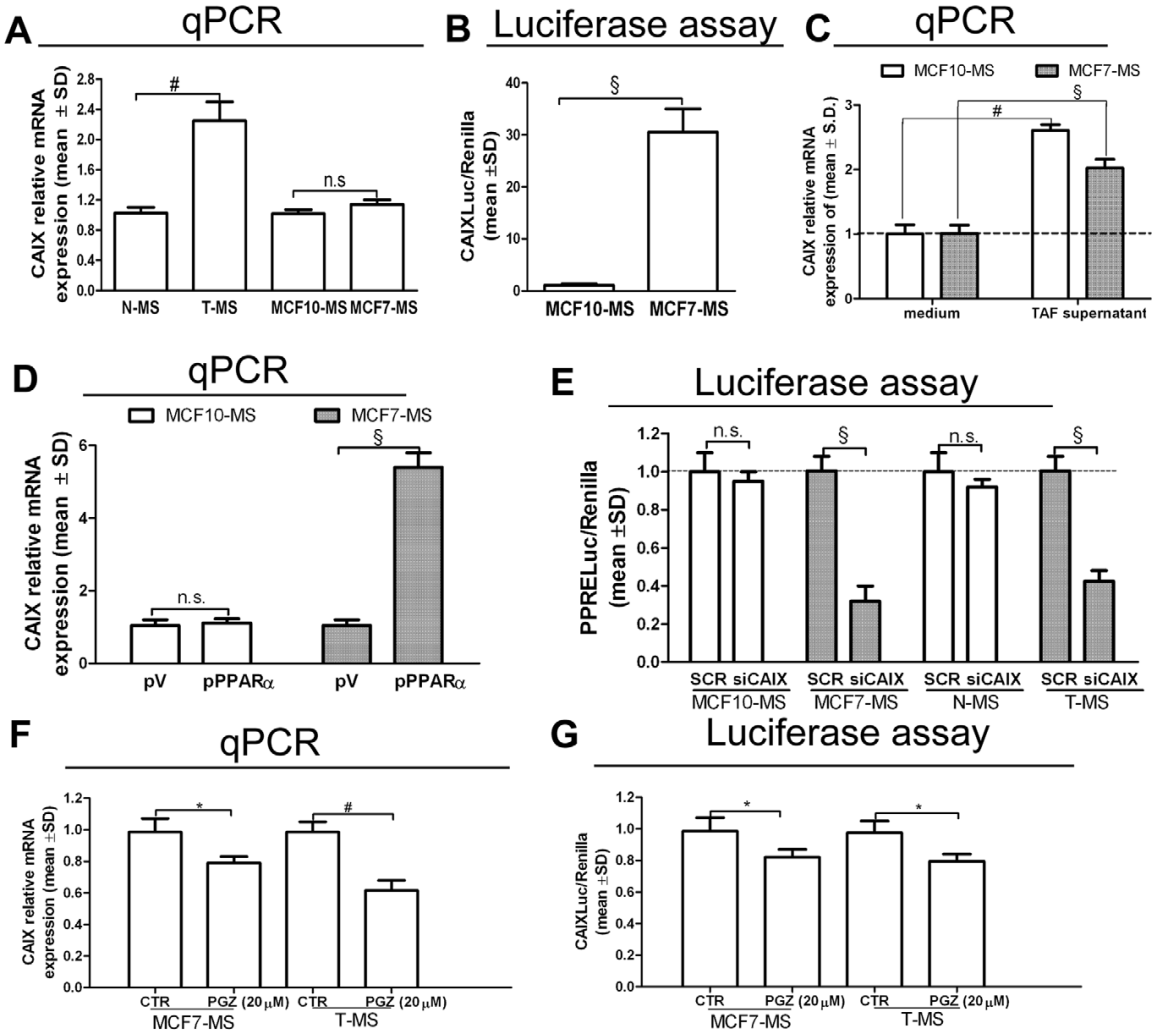
CAIX is under the control of the PPARα/HIF1α interplay in breast CSCs. (A) CAIX mRNA qPCR analysis in MCF10/MCF7-MS and in N/T-MS (samples 1–5, n = 5). (B) CAIXLuc activity in MCF10-MS and MCF7-MS. (C) CAIX mRNA qPCR analysis in TAF supernatant (10%, 24 h)-exposed MCF7-MS and MCF10-MS. (D) CAIX mRNA qPCR analysis in pV/pPPARα.(24 h)-transfected MCF10-MS and MCF7-MS. (E) PPRELuc activity in SCR/siCAIX (72 h)-transfected MCF10/MCF7-MS and N/T-MS (samples 16–18). CAIX mRNA qPCR analysis (F), CAIXLuc activity assay (G) in PGZ (20 µM, 24 h)-exposed MCF7-MS and T-MS (samples 19–20): Data are expressed as mean ±S.D., n = 3 unless otherwise specified, *p<0.05, ^#^ *p*<0.01, ^§^ *p*<0.005, ANOVA test. n.s.: not significant.

CAIX is a tumor antigen and it is an acknowledged HIF1α target [38], also in MCF7-MS, where it is up-regulated by HIF1α over-expression (+93%, *p*<0.01) and down-regulated by HIF1α KD (−46%, *p*<0.01, Figure S7A). We observed that CAIX was expressed to a higher extent in T-MS than in N-MS (Figure 6A), as well as in MCF7-MS than in MCF10-MS (+61%, *p*<0.05, MCF10-MS *vs* MCF10; +45%, *p*<0.05, MCF7-MS vs MCF7, Figure S7B). Accordingly, CAIX promoter activity (CAIXLuc) was higher in MCF7-MS compared to MCF10-MS (Figure 6B). Moreover, the exposure to the TAF supernatant elicited CAIX expression (Figure 6C). We then demonstrated that PPARα over-expression induced the expression of CAIX in MCF7-MS (+63%, *p*<0.01, Figure S7C), but not in MCF10-MS (Figure 6D). Accordingly, siPPARα transfection hampered (−24%, *p*<0.05), as well as the PPARα agonist WY triggered (+25%, *p*<0.05) CAIXLuc activity in MCF7-MS (Figure S7D). Interestingly, the siRNA mediated KD of CAIX (siCAIX) reduced PPRELuc activity in T-MS and MCF7-MS, but neither in N-MS nor in MCF10-MS (Figure 6E). In turn, PGZ reduced CAIX expression (Figure 6F) and CAIXLuc activity (Figure 6G) in MCF7-MS and T-MS. Intriguingly, PGZ reduced CAIX expression (−31%, *p*<0.05) also in TAF (Figure S8). These data point out that the PPARα/HIF1α interplay controls CAIX expression in CSCs.

### ApoE Over-expression in T-MS is Under the Control of the PPARα/HIF1α Interplay

**Figure 7:**
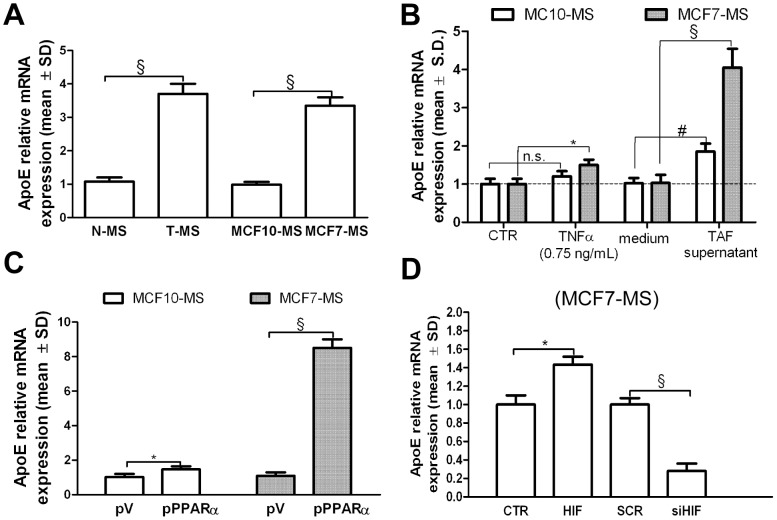
ApoE is under the control the PPARα/HIF1α interplay in breast CSCs. (A) ApoE mRNA qPCR analysis in N−/T-MS (samples 1–5) and in MCF10/MCF7-MS. (B) ApoE mRNA qPCR analysis in TAF supernantants (10%, 24 h), TNFα (0.75 ng/mL, 24 h)-exposed MCF7-MS. ApoE mRNA qPCR analysis in (C) pV/pPPARα (48 h)-transfected MCF7-MS and MCF10-MS, and in (D) HIF1 vector (48 h) or SCR/siHIF1 (72 h)-transfected MCF7-MS. Data are expressed as mean ±S.D., n = 3, *p<0.05, ^#^ *p*<0.01, ^§^ *p*<0.005, ANOVA test. n.s.: not significant.

PPARα is involved in a wide variety of cellular functions, including lipid homeostasis [39]. Moreover, PPRE consensus sequence occurs at the promoters of lipid transporters, such as apolipoproteins [39]. Interestingly, ApoE is over-expressed in MS [7], [24]. Here, we were able to quantify the over-expression of ApoE in T-MS compared to N-MS, as well as in MCF7-MS compared to MCF10-MS (Figure 7A). We also found ApoE over-expression in MCF7-MS and MCF10-MS in response to exogenous TNFα and to the TAF supernatant administration (Figure 7B). Then, we demonstrated that PPARα over-expression induced the mRNA expression (Figure 7C) and protein (+81%, *p*<0.01, Figure S9A) of ApoE in MCF7-MS. As expected, the phenomenon was mimicked by the administration of WY (+127%, *p*<0.005, in MCF10-MS and +135%, *p*<0.005, in MCF7-MS, Figure S9B). Furthermore, HIF1 vector elicited (+44%, *p*<0.05, Figure S9C), as well as siHIF1 reduced the expression of ApoE in MCF7-MS (Figure 7D). These data show that ApoE over-expression in breast cancer stem cells is under the control of the PPARα/HIF1α interplay.

**Figure 8:**
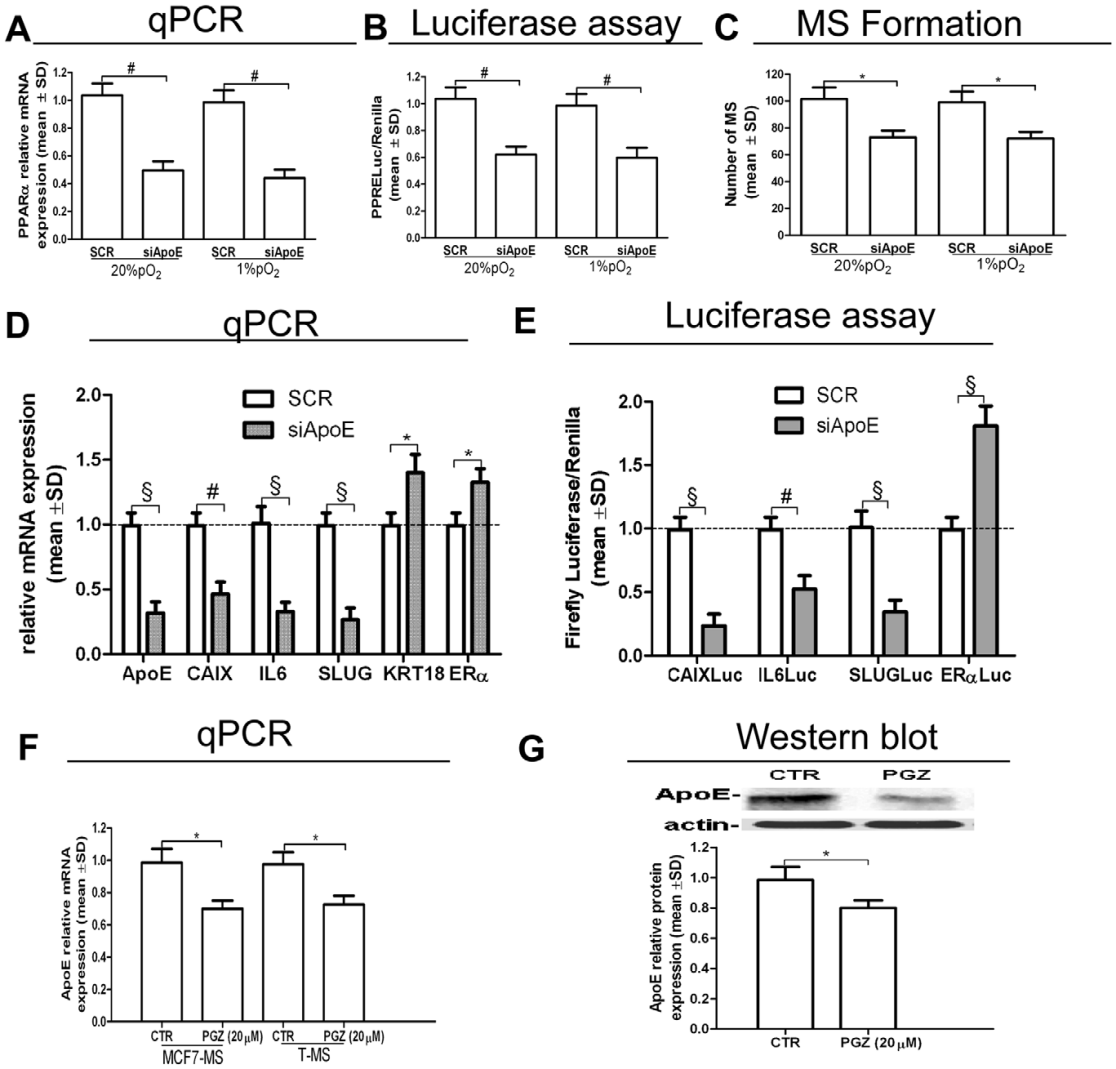
Inhibition of ApoE expression in T-MS. (A) PPARα mRNA qPCR analysis, (B) PPRELuc activity and (C) MS formation assay in siApoE (48 h)-transfected MCF7 cells, in normoxic and hypoxic condition; (D) ApoE CAIX, IL6, SLUG, KRT18 and ERα mRNA qPCR analysis in siApoE (48 h)-transfected MCF7-MS. (E) CAIXLuc, IL6Luc, SLUGLuc and ERαLuc activity in siApoE (48 h)-transfected MCF7-MS; ApoE mRNA qPCR analysis (F) and ApoE protein expression (G) in PGZ (20 µM, 24 h)-exposed MCF7-MS and T-MS (samples 19–20). Data are expressed as mean ±S.D., n = 3, *p<0.05, ^#^ *p*<0.01, ^§^ *p*<0.005, ANOVA test. n.s.: not significant.

We thereafter investigated the effects of the inhibition of ApoE expression in MS. siRNA-mediated (siApoE) KD of ApoE mRNA (−77%, *p*<0.005 in MCF10-MS, −82%, *p*<0.005 in MCF7-MS, Figure S9D) and protein (−69%, *p*<0.01, in MCF7-MS, Figure S9E) reduced PPARα expression, PPRELuc activity and MCF7-MS formation capability (Figure 8A, B, C). ApoE KD also elicited the expression of the differentiation markers keratin-18 (KRT18) and ERα, and reduced the expression (Figure 8D) and the promoter activity (Figure 8E) of CAIX, IL6 and SLUG genes. Consistent with the expectations was the finding that PGZ hindered ApoE mRNA expression in MCF7-MS and T-MS (Figure 8F) and ApoE protein expression in MCF7-MS (Figure 8G), but not in MCF10-MS (Figure S10). These data point at the role of the PPARα/HIF1α interplay in the regulation of ApoE in CSCs.

## Discussion

Our investigation started with the observation that human models for breast CSCs (T-MS and MCF7-MS) display increased TNFR1 compared to their normal/non tumorigenic counterparts (N-MS and MCF10-MS). Breast CSCs also exhibit an increased growth response to the TAF supernatant. Though TAF secrete low amounts of TNFα, we demonstrate that TAF elicit TNFα expression in CSCs by secreting TGFβ, thus setting up an autocrine TNFα loop that enhances MS growth. TNFR1 signalling is a major inducer of inflammatory response via NF-κB activation [40]–[43]. The expression of the two NF-κB targets TNFα and IL6 is higher in CSCs, and both these cytokines have been previously shown to elicit MS formation [12]–[13], [44], [45]. Collectively, the findings here reported, together with previous data concerning the increase of NF-κB activity in T-MS [24], contribute to the tenet that CSCs are endowed with pro-inflammatory phenotype [14], [16], [18].

We then pinpoint that the core of such TAF-promoted pathway involves the PPARα/HIF1α interplay. In particular, the two proteins induce each other expression and trigger T-MS growth to a higher extent than their normal counterpart. In this regard, we show the involvement of miR130b. Over-expression of this microRNA has been previously reported in liver cancer and pluripotent stem cells [46], [47]. Moreover, miR130b is induced by hypoxia and increases HIF1α protein expression by facilitating HIF1α mRNA translation, via the down-regulation of DDX6 expression [36]. Here, we report that miR130b up-regulates HIF1α activity (as well as down-regulates DDX6 expression), and that the up-regulation of the PPARα/HIF1α interplay is paralleled by the down-regulation of PPARγ, a miR130b target [37]. In regard to this issue, we provide evidence that the PPARγ agonist PGZ, which up-regulates PPARγ expression and activity [24], hinders the PPARα/HIF1α interplay in breast CSCs. We propose that this phenomenon is mediated by miR17-5p which targets both PPARα and HIF1α mRNA 3′UTRs [48]. With respect to this issue, we show that miR17-5p expression is up-regulated by PGZ and that miR17-5p knock-down increases PPARα expression, concomitantly with the reduction of PPARγ expression. Thus, miR17-5p may mediate the PPARα/HIF1α interplay switch-off throughout the induction of PPARγ over-expression. Interestingly, miR17-5p has been previously found to be repressed by hypoxia [49]. Nevertheless, miR17-5p has been reported as a pro or anti oncogenic miR depending upon the genetic and environmental context [50].

We identified two acknowledged regulators of breast CSCs, namely IL6 and SLUG as targets of the PPARα/HIF1α interplay. The former has been characterized as crucial mediator of breast CSCs growth capacity *in vitro* [10]. The latter was recently demonstrated to play a pivotal role in normal and tumor mammary gland self renewal in human and mice [5], [12]. We however focussed our attention on two additional targets of the PPARα/HIF1α interplay. The former target is the hypoxia inducible gene CAIX, which we found over-expressed in T-MS, in compliance with its original definition as tumor antigen [51]. We previously reported that CAIX expression is crucial for MS hypoxia survival [9], [22]. Other investigations showed that CAIX sustains breast cancer survival and invasive behaviour [52]. CAIX is an HIF1α target and contributes to cancer aggressiveness in various biological contexts, such as the basal-like breast tumor subtype [12], [53]. The data here presented lead to hypothesize that CAIX over-expression in CSCs may be the consequence of cues that pertain to the cancer stem cell niche. These data encourage to pursue the ongoing research on CAIX inhibitory molecules as anti cancer agents [54].

The latter target controlled by the PPARα/HIF1α interplay is ApoE, a major component of circulating lipoproteins [55]. Here we found the over-expression of ApoE in T-MS, recalling a similar finding in prostate CSCs [14]. We then demonstrate that ApoE knock-down reduces MS formation and the expression of CAIX, IL6 and SLUG [5], [12]. These data agree on the role of ApoE in breast cancer aggressiveness. In fact, ApoE plays a crucial role in human pathology, as the ε4 allele represents a frailty variant that predisposes to various age-related diseases [56]. ApoE knock-out mice disclose increased mammary tumor incidence, likely in relationship with their hyperlipidemic state [57]. Intriguingly, ApoE physically interacts with HCCR-1, an onco-protein that promotes breast cancer [58]. However, the relationship between breast cancer and ApoE in humans is still under debate, and ApoE may impact disease susceptibility or response to therapy [59], [60]. Interestingly, whereas ApoE is likely to impact cardiovascular diseases due to an alteration of the circulating lipidic profile, its role in cancer seems to be independent of this association [61].

**Figure 9:**
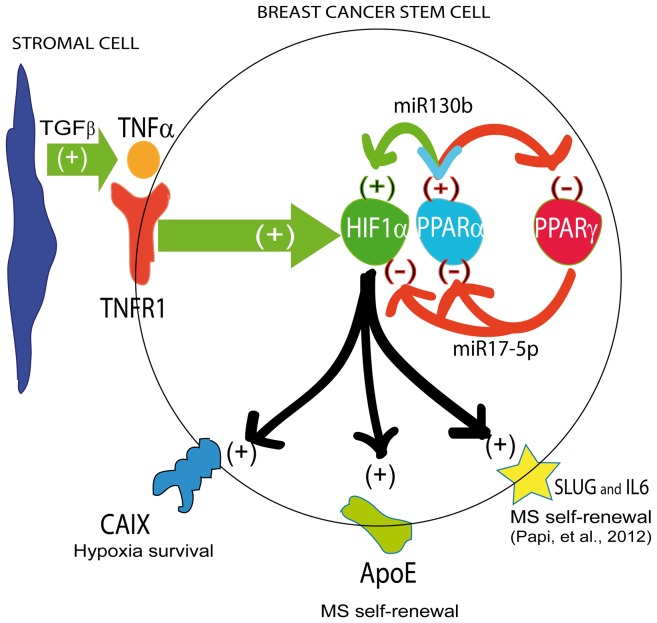
Schematic representation of the data. TAF secreted TGFβ induces TNFα expression in breast CSCs. TNFα binds TNFR1 on breast CSCs and activates the PPARα/HIF1α interplay which up-regulates miR130b expression. The interplay is counterbalanced by PPARγ via miR17-5p up-regulation. In turn, the PPARα/HIF1α interplay regulates CAIX, ApoE, IL6 and SLUG expression.

In conclusion, we show that the PPARα/HIF1α interplay, triggered in breast CSCs by the tumor associated fibroblast secreted TGFβ, engenders the expression of two acknowledged breast CSCs regulatory genes (IL6 and SLUG), as well as up-regulates two less characterized regulators of breast CSCs, namely CAIX and ApoE (Figure 9). This molecular machinery is counter-acted by PPARγ expression. Our data lead to envisage the possibility to harness nuclear receptor regulation of pro-inflammatory pathways to negatively interfere with CSCs survival.

## Materials and Methods

### Chemicals

Recombinant human IL6, TGFβ, TNFα, and anti-human TNFα inhibitory antibody were purchased from Sigma (St Louis, MO, USA) and dissolved in phosphate buffered saline (1 mg/mL). The PPARγ agonist PGZ (Alexis, Lausen, Switzerland) and the PPARα agonist WY (Sigma) were dissolved in dimethylsulfoxide (0.1 M).

### Plasmids, microRNA and siRNA Transient Transfection

PPARα (pPPARα) and empty (pV) plasmids were obtained from Institute Pasteur (Lille, France). HIF1 plasmid was obtained from Eric Huang (Department of Neurosurgery, University of Utah, Salt Lake City, Utah, USA). PPARα and ApoE specific double-strand RNA oligonucleotides (siRNA) and appropriate scramble siRNA (SCR) were purchased from Origene (Rockville, MD, USA). CAIX and HIF1 siRNA and appropriate control SCR siRNAs were purchased from Invitrogen (Rockville, MD, USA). Pre-miRNA17-5p, miRNA130b, antago-miRNA17-5p and miRNA130b were purchased from Life Technologies (Rockville, MD, USA).

### Cell Cultures and Generation of MCF7 and MCF10 MS

MCF7 were grown in RPMI 1640 medium supplemented with 10% FBS, penicillin-streptomycin and glutamine. MCF10 [62] were grown in DMEM medium with 20% FBS, supplemented with 10 µg/ml insulin, 10 µM hydrocortisone and 10 µg/ml EGF. Hypoxia (1% pO_2_) was generated in an Vivo^2^300 hypoxic workstation (Ruskinn Technologies, Ireland). MCF7-MS and MCF10-MS were generated by plating 2500 cells into 3-cm^2^ low-attachment wells (Corning, NY, USA) in mammary epithelial growth medium (MEGM), supplemented with B27, 10 ng/ml epidermal growth factor (EGF), 10 ng/ml basic fibroblast growth factor (bFGF), 10 µg/ml insulin, 10^−6^ M hydrocortisone (Voden Medical, Rome, Italy). Primary MS formation usually occurs after 48 to 72 h. To examine the effects of chemicals on MS formation and MS gene expression, MCF7-MS and MCF10-MS were exposed to each molecule and assessed after 24 h to 72 h. MS with an apparent diameters ≥50 µm were scored and photographed using a inverted microscope (Olympus CKX41, digital cameras Olympus C-5060, Japan). To examine the impact of each specific expression vector, siRNA or pre/antago-miR on MS formation and MS gene expression, adherent MCF7 or MCF10 (10^5^ cells in a 3-cm^2^ well) were transfected with 1 µg/well of each siRNA or pre/antago-miR using Lipofectamine 2000 (Invitrogen, USA). After 6 h of incubation, cells were re-suspended, seeded in 24-well ultra-low attachment plates at a density of 2500 cells per well and assessed after 48 h or 72 h. The Effect of each specific treatment was determined by at least n = 3 independent experiments. MDA-MB231 cells were grown in RPMI 1640 medium supplemented with 10% FBS, penicillin-streptomycin and glutamine.

### Generation of MS from Normal and Breast Carcinoma Human Tissues

Twenty-two fresh surgical specimens, obtained from patients with ductal breast carcinoma who underwent quadrantectomy or mastectomy, were collected for the study (Table S1). Normal and tumor samples were processed as previously described [9], [12]. Briefly, tissues were placed in sterile Epicult (Voden Medical), minced with sterile scalpels, and incubated for 6–12 h in the presence of 1000 U Collagenase/Hyaluronidase enzyme mix (Voden Medical). Samples were centrifuged at 80×g, and the pellet was digested by Dispase and DNAse (Voden Medical), and then pelleted at 450×g. Pellets were re-suspended, filtered through a 40-µM nylon mesh (Voden Medical), and plated into 3-cm^2^-well low attachment plates (Corning, NY, USA), filled with 3 ml MEGM, supplemented with B27, 10 ng/ml EGF, 10 ng/ml bFGF, 10 µg/ml insulin, 10^−6^ M hydrocortisone (Voden Medical). Primary MS started forming after 4–6 days and were processed at day 14. Self renewal of MS was tested by assessing the capacity of primary MS to generate secondary MS after trypsin disaggregation. Transfection in primary T-MS was performed by mixing 1 µg of each expression vector or siRNA with in vitro JET-PEI reagent (Poly-plus transfection, USA). The procedure was approved by the local ethical committee of Center for Applied Biomedical Research, St. Orsola-Malpighi University Hospital (Bologna, Italy) (Prot n.75/2011) and by the patients’ written informed consent.

### Isolation of Fibroblasts from Normal and Tumour Breast Tissues and Collection of the Fibroblasts Supernatant

Fibroblasts were collected by centrifuging the Collagenase/Hyaluronidase digested tissue lysates used for MS generation at 500×g for 5 min (see above). The fibroblasts containing pellet was re-suspended and cultured in DMEM medium with 20% fetal bovine serum (FBS, Euroclone, Milan, Italy), penicillin-streptomycin and glutamine (Sigma), in 6-well plates. When the fibroblasts reached confluence, medium was discarded and replaced with fresh medium, containing DMEM+FBS 0.5% (1.5 mL/well), for 24 h. Supernatants were then collected, centrifuged for 5 min at 10^5^×g to remove debris and conserved at −80°C. For the experimental setting supernatants were diluted in MS medium (MEGM) at the final concentration of 10%, and already formed MS were exposed for 24–72 h.

### RNA Extraction, Real-time Reverse Transcription Quantitative PCR (qPCR) and Reverse Transcription PCR (RT-PCR)

Total RNA was extracted from cultured cells, MS and fibroblasts using TRIzol (Life Technologies, Rockville, MD, USA) reagent following the customer’s instructions. Real-time Reverse Transcription quantitative PCR (qPCR) analysis was performed by TaqMan approach in a Gene Amp 7000 Sequence Detection System (Life Technologies, Rockville, MD, USA), as previously described [24]. Each sample was analyzed in replicates (n = 3). Sets of primers and fluorogenic probes specific for the target genes (Table S2) were purchased from Applied Biosystems; qPCR conditions are: pre-denaturation step at 95°C for 2 min; 28 cycles of denaturation at 95°C for 1 min, annealing at the appropriate temperature for 1 min, extension at 72°C for 1 min; final extension at 72°C for 7 min. Human beta-glucuronidase was used as an endogenous control for mRNA level. 6 URNP was used as an endogenous control for miRNA level. The relative amount of each target mRNA or miRNA was calculated as: N target 2^− (DCt sample−DCt calibrator)^, where DCt values of the sample and calibrator were determined by subtracting the Ct value of the endogenous control gene from the Ct value of each target gene. RT-PCR analysis was performed using the Master RT plus PCR system kit according to the instruction of the supplier (Life Technologies). Actin was used as an internal control. RT-PCR was performed for 31 cycles (1 minute/annealing) for each primer, except for CAIX that was performed for 34 cycles. Primer sequence and PCR parameters are reported in Table S3.

### Luciferase Assay

SLUGLuc, containing the −800/+10 bp SLUG promoter sequence in the pGL3 basal vector, was kindly provided by Dr. Togo Ikuta (Saitama Cancer Centre, Saitama, Japan). ERαLuc plasmid, which contains 3 copies of estrogen response element (ERE), was kindly provided by Dr. Rakesh Kumar (Department of Molecular and Cellular Oncology, MD Anderson Cancer Center, Houston, Texas). CAIXLuc, containing the −179/+34 bp promoter sequence of CAIX, was provided by J Pastorek (Slovak academy of science, Bratislava). Hypoxia responding element (HRE-Luc), containing 3 copies of HIF1 consensus was kindly provided by Dr. Giovanni Melillo (Tumor hypoxia laboratory, National Cancer Institute, Frederick, MD, USA). IL6Luc, containing the −2161 to −41 bp IL6 promoter sequence, was kindly provided by Dr. WL Farrar (NCI-Frederick Cancer Research and Development Center, Frederick, MD, USA). PPRELuc, containing 7 copies of PPARs consensus, was kindly provided by Professor Ronald Evans (Salk Institute, La Jolla, CA). NF-κBLuc was previously described [12]. Each of the above plasmids (1 µg) were co-transfected with a thymidine kinase promoter driven Renilla luciferase (400 ng) plasmid as a reference control (Promega, USA). MS transfection was performed with JET-PEI reagent (Poly-plus transfection) (3 µL for 1 µg plasmid) and Luciferase activity was assayed after 48 h using the Dual-Luciferase® Reporter Assay System (Promega), according to the manufacturer’s instructions. Luciferase activity was normalized over Renilla activity and all reported experiments were performed in triplicates.

### Western-blot Analysis (WB)

Cell lysates were prepared, run, and blotted as previous described [24] and probed with specific antibodies: rabbit polyclonal anti-PPARγ (Pierce, Rockford, MD, USA), mouse monoclonal anti-ApoE (Origene, Rockville, MD, USA), anti-PPARα (Thermoscientific, Rockford, MD, USA), anti-CAIX (clone M-75, kindly provided by Jaromir Pastorek, Slovak academy of science, Bratislava), anti-HIF1α (Pierce, USA), anti-actin (SantaCruz, USA). Protein levels were detected by direct acquisition of chemiluminescence in an imager (ChemiDoc XRS, Bio-Rad, Milan, Italy) by luminol (Millipore, USA) and were quantified in triplicates using a densitometric image analysis software (Quantity One 4.6, Bio-Rad, Milan, Italy).

### Elisa Test

Determination of TNFα and TGFβ level in TAF and NAF supernatant were evaluated by ELISA (S.I.C., Rome, Italy). Briefly, cells were seeded in a 6-well plate at the density of 3×10^5^ cells per well and collected in serum-free medium for 24 h. The harvested medium was centrifuged at 500×g for 5 min (4°C) to remove floating cells and the supernatants were collected and assayed following the customer’s instructions.

### Statistical and Bioinformatic Analysis

Statistical significance was assessed by ANOVA followed by Bonferroni’s multiple comparison test or two-tail Student’s t-test, as appropriate, using PRISM 5.1 (Graphpad Software, La Jolla, CA, USA). The level for accepted statistical significance is *p*<0.05. mRNA 3′-UTR were analyzed for miRNA binding site by the on-line software Targetscan (www.Targetscan.com).

## Supporting Information

Figure S1 **TNFα and the TAF supernatant induce Jagged1 expression in MS.** Jagged1 mRNA qPCR analysis in MCF10/MCF7-MS and in N−/T-MS (samples 14–15) exposed to (A) TNFα (0.75 ng/mL, 24 h). (B) WB analysis of PPARα protein level in TAF supernatant (10%, 24 h)-exposed MCF7-MS. (C) Jagged1 mRNA qPCR analysis in MCF10/MCF7-MS and in N−/T-MS (samples 14–15) exposed to NAF and TAF supernatant (10%, 24 h). Data are expressed as mean ±S.D., n = 3, *p<0.05, ^#^ *p*<0.01, ^§^ *p*<0.005, ANOVA test.
(TIF)

Figure S2 **Hypoxia and the TAF supernatant induce HIF1α activity and expression in MS.** (A) HIF1α mRNA RT-PCR and WB analysis in TAF supernatant (10%, 24 h)-exposed normoxic/hypoxic MCF7 and MCF10. HIF1α mRNA RT-PCR analysis (B) and HRELuc activity (C) in normoxic and hypoxic MCF10-MS and MCF7-MS. (D) PPARα mRNA qPCR analysis in SCR/siPPARα (72 h)-transfected normoxic and hypoxic MCF7-MS. (E) HIF1α protein WB analysis in SCR/siPPARα (72 h)-transfected and WY (10 µM, 24 h)-exposed MCF10-MS. Data are expressed as mean ±S.D., n = 3, *p<0.05, ^#^ *p*<0.01, ^§^ *p*<0.005, ANOVA test. n.s.: not significant.
(TIF)

Figure S3 **The PPARα/HIF1 interplay regulates SLUG and IL6 in MS.** SLUG (A) and IL6 (B) qPCR mRNA analysis in pV/pPPARα transfected MCF7-MS and MCF10-MS (48 h). SLUG (C) and IL6 (D) qPCR analysis in HIF1 (48 h) or SCR/siHIF1 (72 h)-transfected MCF7-MS. Data are expressed as mean ±S.D., n = 3, *p<0.05, ^#^ *p*<0.01, ANOVA test.
(TIF)

Figure S4 **Nuclear receptors expression are regulated by HIF1 in MS.** (A) PPARγ WB analysis in HIF (24 h)-transfected MCF7-MS. (B) PPARα and PPARγ WB analysis in MCF10, MCF10-MS, MCF7 and MCF7-MS cells. (C) PPARβ, RXRα, RXRβ and RXRγ mRNA RT-PCR analysis in MCF10 and MCF10-MS cells. (D) HIF1α, PPARβ, RXRα, RXRβ and RXRγ mRNA RT-PCR analysis in HIF1 (24 h) and SCR/siHIF1 (72 h)-transfected MCF7-MS. Data are expressed as mean ±S.D., n = 3, *p<0.05, ^#^ *p*<0.01, ^§^ *p*<0.005, ANOVA test. n.s.: not significant. n.d.: not detected.
(TIF)

Figure S5 **Effects of PPARγ agonist (PGZ) on CSCs pro-inflammatory pathways and on MS formation.** (A) HIF1α mRNA RT-PCR analysis, (B) number of MS and (C) qPCR analysis of IL6, Notch3, Jagged1 mRNA levels in PGZ (20 µM, 24 h)-exposed hypoxic MCF7-MS and T-MS (samples 18–20). Data are expressed as mean ±S.D., n = 3 *p<0.05, ^#^ *p*<0.01, ^§^ *p*<0.005, ANOVA test. n.s.
(TIF)

Figure S6 **Effects of PGZ on CSCs pro-inflammatory pathways in MDA-MB-231 cells.** IL6, IL8, SLUG and TNFα, mRNA RT-PCR analysis in PGZ (20 µM, 24 h)-exposed MDA-MB-231 breast cancer cells. Data are expressed as mean ±S.D., n = 3, *p<0.05, ^#^ *p*<0.01, ANOVA test.
(TIF)

Figure S7 **CAIX expression is regulated by PPARα/HIF1 interplay in MS.** (A) CAIX mRNA qPCR analysis in HIF1 vector (24 h) or SCR/siHIF1 (72 h)-transfected MCF7-MS. (B) WB analysis of CAIX protein expression in hypoxia exposed MCF10, MCF10-MS, MCF7 and MCF7-MS (C), and in pV/pPPARα (24 h)-transfected MCF7-MS (D). CAIXLuc assay SCR/siPPARα (72 h)-transfected and WY (10 µM, 24 h)-exposed hypoxic MCF7-MS. Data are expressed as mean ±S.D., n = 3, *p<0.05, *^#^p*<0.01, ANOVA test.
(TIF)

Figure S8 **Effects of PGZ on CAIX expression in TAF.** CAIX mRNA RT-PCR analysis in PGZ (20 µM)-exposed hypoxic TAF (24 h, samples 21–22). Data are expressed as mean ±S.D., *p<0.05, ANOVA test.
(TIF)

Figure S9 **ApoE expression is regulated by PPARα in MS.** (A) WB analysis of ApoE protein expression in pV/pPPARα (24 h)-transfected MCF7-MS. (B) ApoE mRNA qPCR analysis in WY (10 µM, 24 h)-exposed MCF10-MS and MCF7-MS. (C) WB analysis of ApoE protein expression in HIF1 vector (48 h)-transfected MCF7-MS. (D) ApoE mRNA qPCR analysis in SCR/siApoE (48 h)-transfected MCF10-MS and MCF7-MS. (E) WB analysis of ApoE protein expression in SCR/siApoE (48 h)-transfected MCF7-MS. Data are expressed as mean ±S.D., n = 3, *p<0.05, ^#^ *p*<0.01, ^§^ *p*<0.005, ANOVA test.
(TIF)

Figure S10 **Effects of PGZ on ApoE expression in MCF10-MS.** (A) ApoE mRNA qPCR analysis and (B) WB analysis of ApoE protein in PGZ (20 µM, 24 h)-exposed MCF10-MS. Data are expressed as mean ±S.D., n = 3, n.s.: not significant.
(TIF)

Table S1 **Clinical–pathological parameters of 22 breast carcinomas used for T-MS, N-MS and fibroblasts isolation.** List of samples used with clinical and pathological parameters. Abbreviations: pT, tumor size; pN, nodal involvement; G, grade; NG, nuclear grade; ER, estrogen receptor; HER-2, ERbB2 kinase receptor; EGFR, epidermal growth factor receptor.
(DOC)

Table S2 **List of probes used in qPCR analysis.**
(DOC)

Table S3 **List of primers used in RT-PCR analysis.**
(DOC)
