# Supplementary figures and images for: Peroxisome Proliferator Activated Receptor-α/Hypoxia Inducible Factor-1α Interplay Sustains Carbonic Anhydrase IX and Apoliprotein E Expression in Breast Cancer Stem Cells
Source: PLoS One. 2013 Jan 25;8(1):e54968. doi: 10.1371/journal.pone.0054968 (PMC3556000; doi:10.1371/journal.pone.0054968)

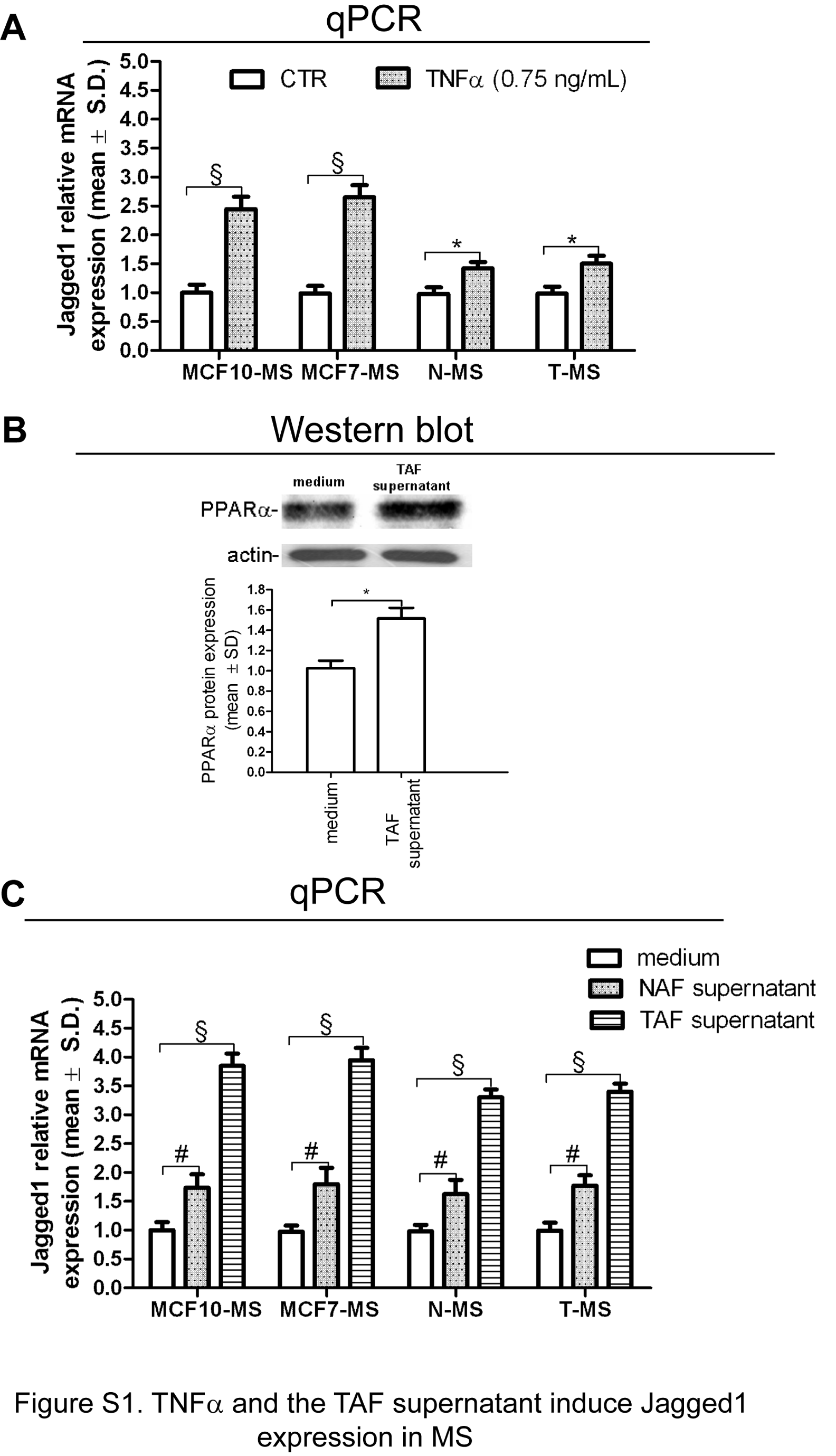

Supplement: Figure S1 — TNFα and the TAF supernatant induce Jagged1 expression in MS. Jagged1 mRNA qPCR analysis in MCF10/MCF7-MS and in N−/T-MS (samples 14–15) exposed to (A) TNFα (0.75 ng/mL, 24 h). (B) WB analysis of PPARα protein level in TAF supernatant (10%, 24 h)-exposed MCF7-MS. (C) Jagged1 mRNA qPCR analysis in MCF10/MCF7-MS and in N−/T-MS (samples 14–15) exposed to NAF and TAF supernatant (10%, 24 h). Data are expressed as mean ±S.D., n = 3, *p<0.05, # p<0.01, § p<0.005, ANOVA test. (TIF) [file pone.0054968.s001.tif]

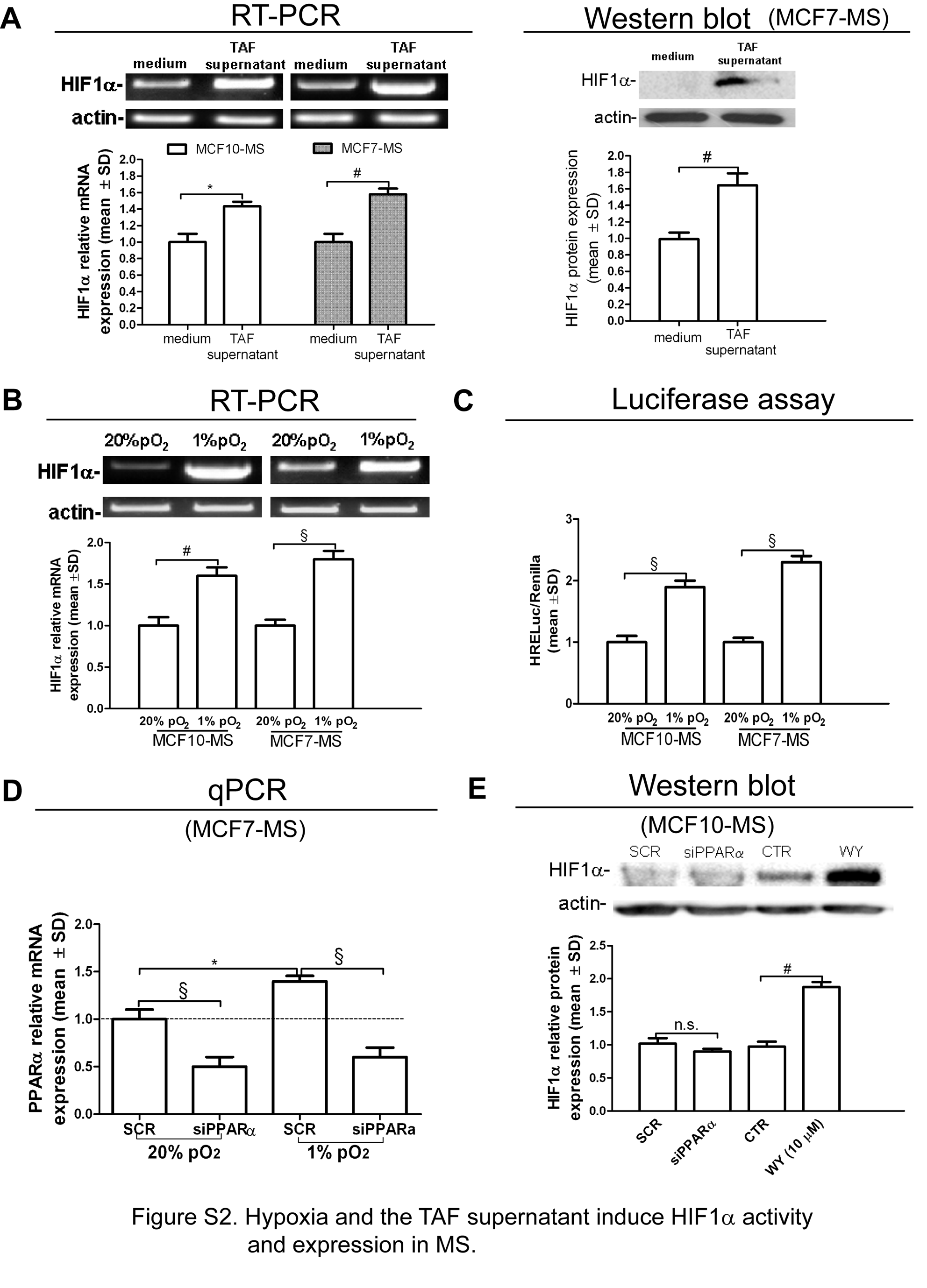

Supplement: Figure S2 — Hypoxia and the TAF supernatant induce HIF1α activity and expression in MS. (A) HIF1α mRNA RT-PCR and WB analysis in TAF supernatant (10%, 24 h)-exposed normoxic/hypoxic MCF7 and MCF10. HIF1α mRNA RT-PCR analysis (B) and HRELuc activity (C) in normoxic and hypoxic MCF10-MS and MCF7-MS. (D) PPARα mRNA qPCR analysis in SCR/siPPARα (72 h)-transfected normoxic and hypoxic MCF7-MS. (E) HIF1α protein WB analysis in SCR/siPPARα (72 h)-transfected and WY (10 µM, 24 h)-exposed MCF10-MS. Data are expressed as mean ±S.D., n = 3, *p<0.05, # p<0.01, § p<0.005, ANOVA test. n.s.: not significant. (TIF) [file pone.0054968.s002.tif]

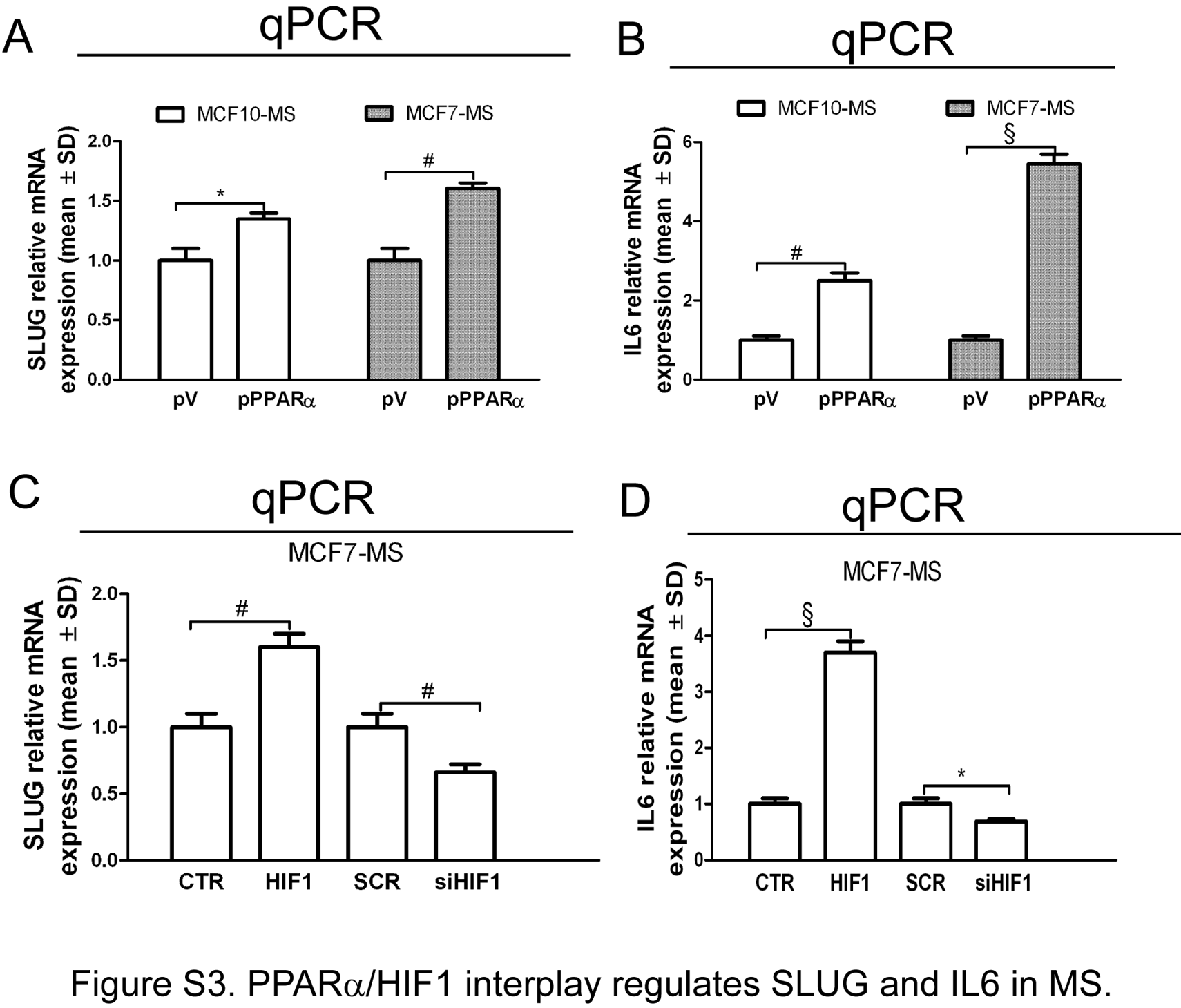

Supplement: Figure S3 — The PPARα/HIF1 interplay regulates SLUG and IL6 in MS. SLUG (A) and IL6 (B) qPCR mRNA analysis in pV/pPPARα transfected MCF7-MS and MCF10-MS (48 h). SLUG (C) and IL6 (D) qPCR analysis in HIF1 (48 h) or SCR/siHIF1 (72 h)-transfected MCF7-MS. Data are expressed as mean ±S.D., n = 3, *p<0.05, # p<0.01, ANOVA test. (TIF) [file pone.0054968.s003.tif]

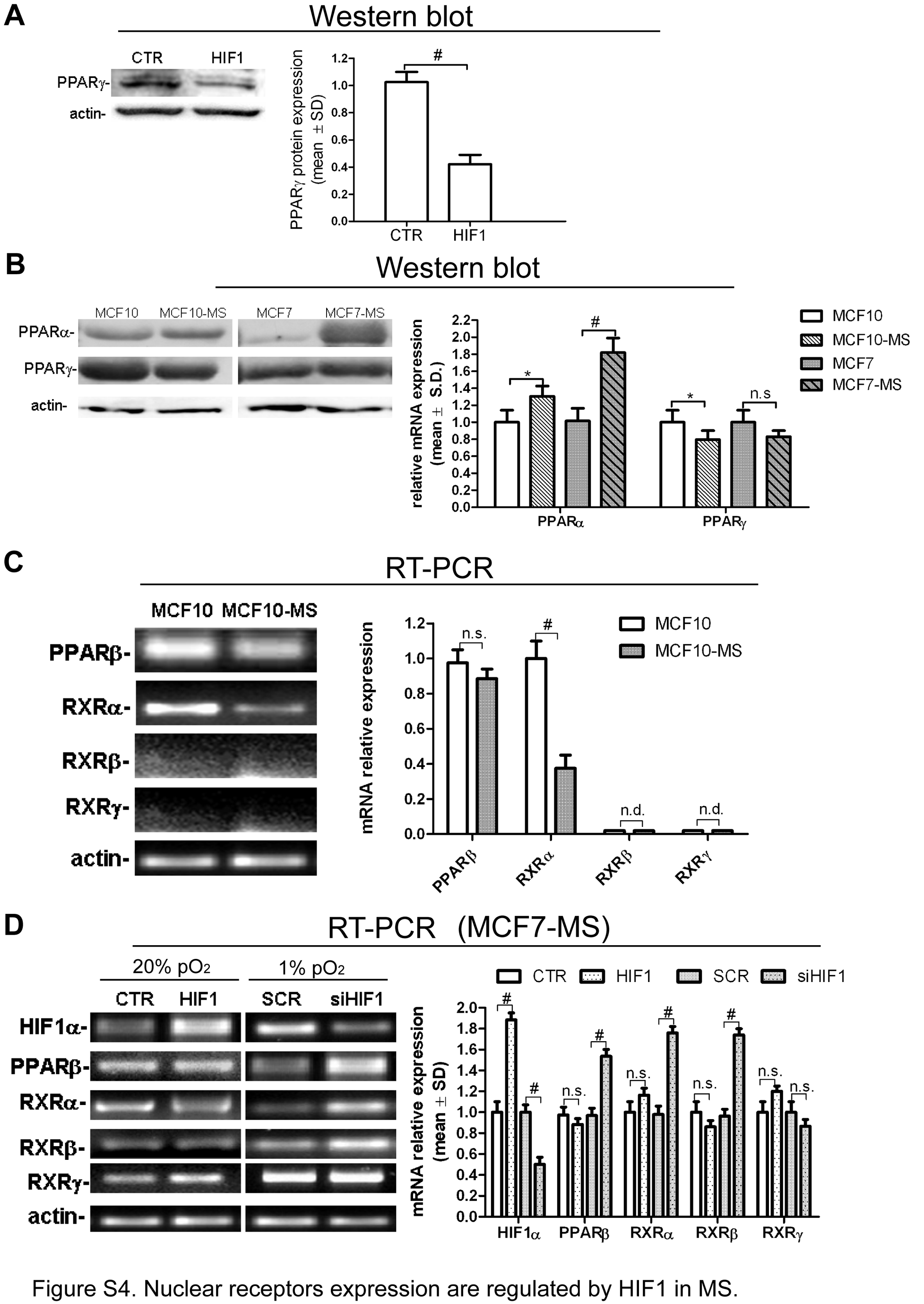

Supplement: Figure S4 — Nuclear receptors expression are regulated by HIF1 in MS. (A) PPARγ WB analysis in HIF (24 h)-transfected MCF7-MS. (B) PPARα and PPARγ WB analysis in MCF10, MCF10-MS, MCF7 and MCF7-MS cells. (C) PPARβ, RXRα, RXRβ and RXRγ mRNA RT-PCR analysis in MCF10 and MCF10-MS cells. (D) HIF1α, PPARβ, RXRα, RXRβ and RXRγ mRNA RT-PCR analysis in HIF1 (24 h) and SCR/siHIF1 (72 h)-transfected MCF7-MS. Data are expressed as mean ±S.D., n = 3, *p<0.05, # p<0.01, § p<0.005, ANOVA test. n.s.: not significant. n.d.: not detected. (TIF) [file pone.0054968.s004.tif]

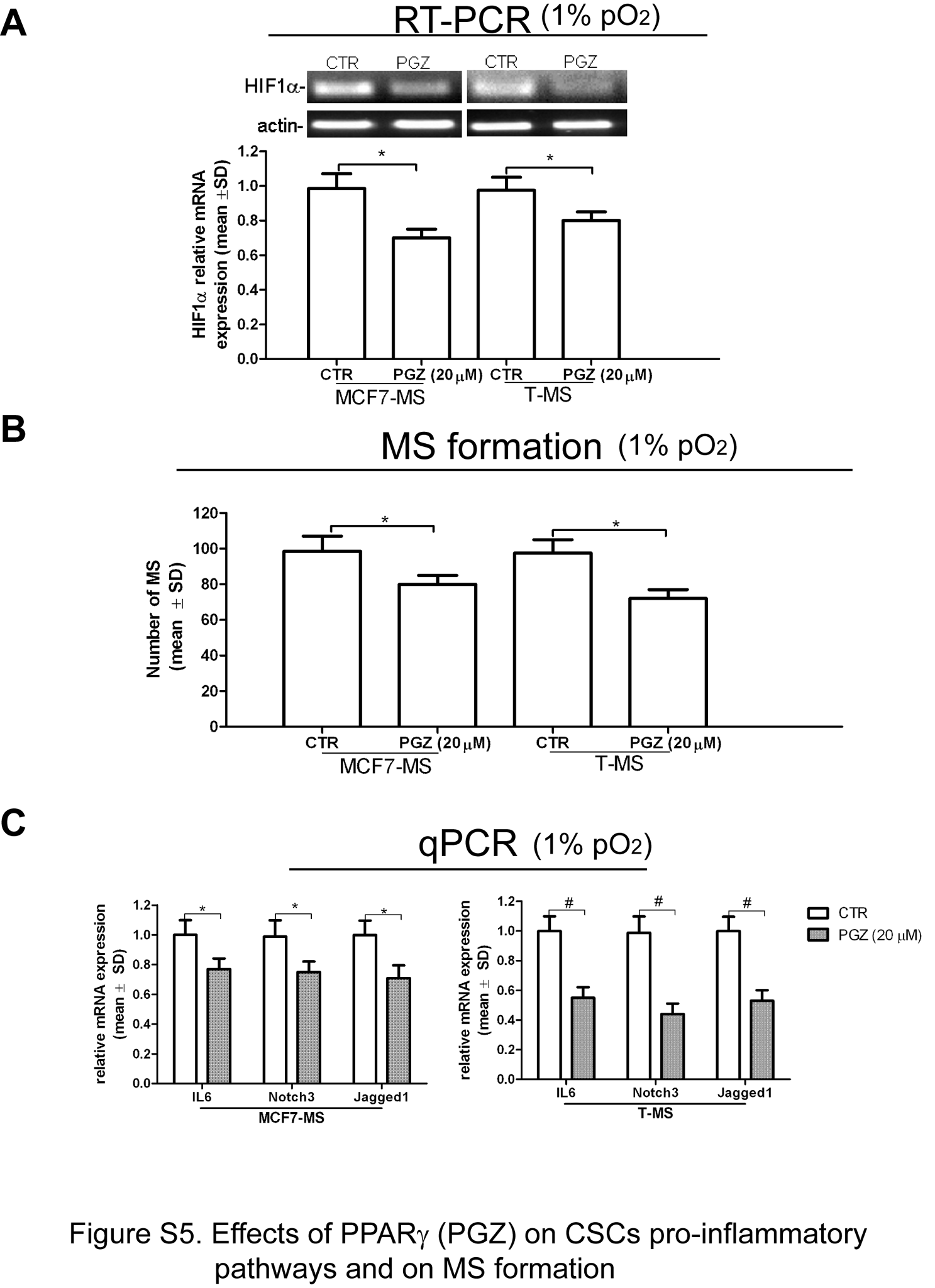

Supplement: Figure S5 — Effects of PPARγ agonist (PGZ) on CSCs pro-inflammatory pathways and on MS formation. (A) HIF1α mRNA RT-PCR analysis, (B) number of MS and (C) qPCR analysis of IL6, Notch3, Jagged1 mRNA levels in PGZ (20 µM, 24 h)-exposed hypoxic MCF7-MS and T-MS (samples 18–20). Data are expressed as mean ±S.D., n = 3 *p<0.05, # p<0.01, § p<0.005, ANOVA test. n.s. (TIF) [file pone.0054968.s005.tif]

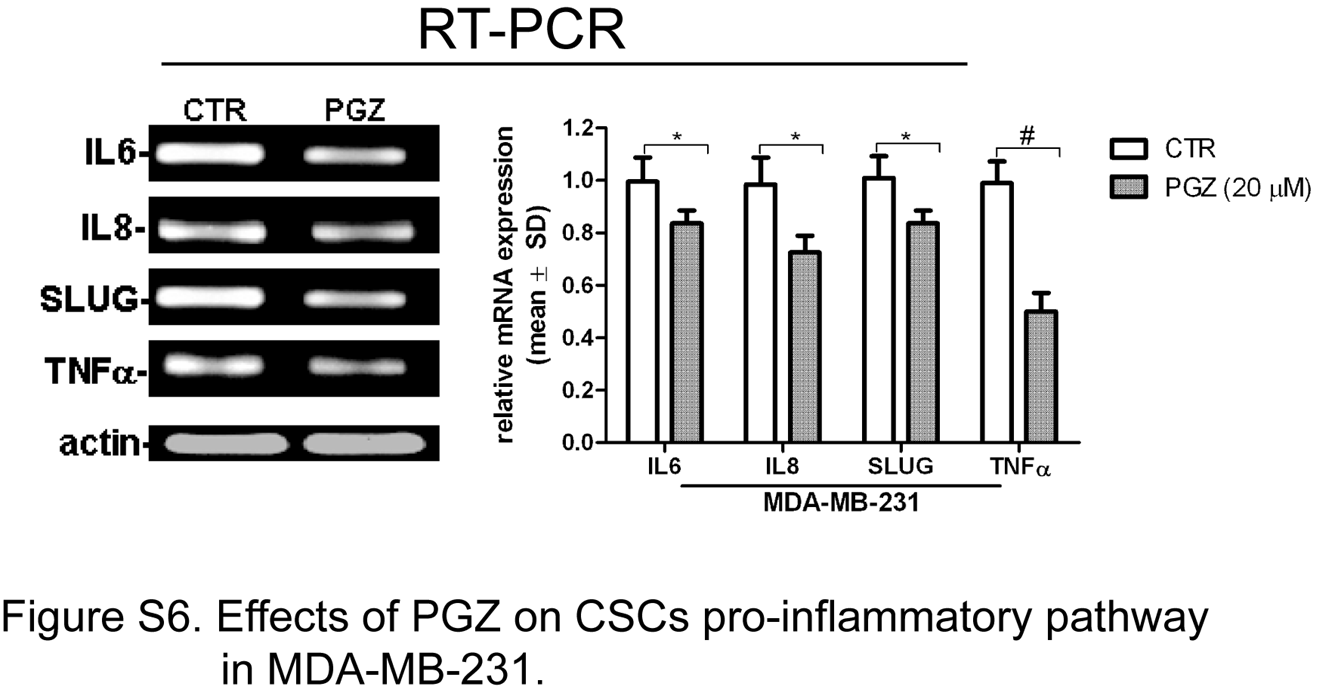

Supplement: Figure S6 — Effects of PGZ on CSCs pro-inflammatory pathways in MDA-MB-231 cells. IL6, IL8, SLUG and TNFα, mRNA RT-PCR analysis in PGZ (20 µM, 24 h)-exposed MDA-MB-231 breast cancer cells. Data are expressed as mean ±S.D., n = 3, *p<0.05, # p<0.01, ANOVA test. (TIF) [file pone.0054968.s006.tif]

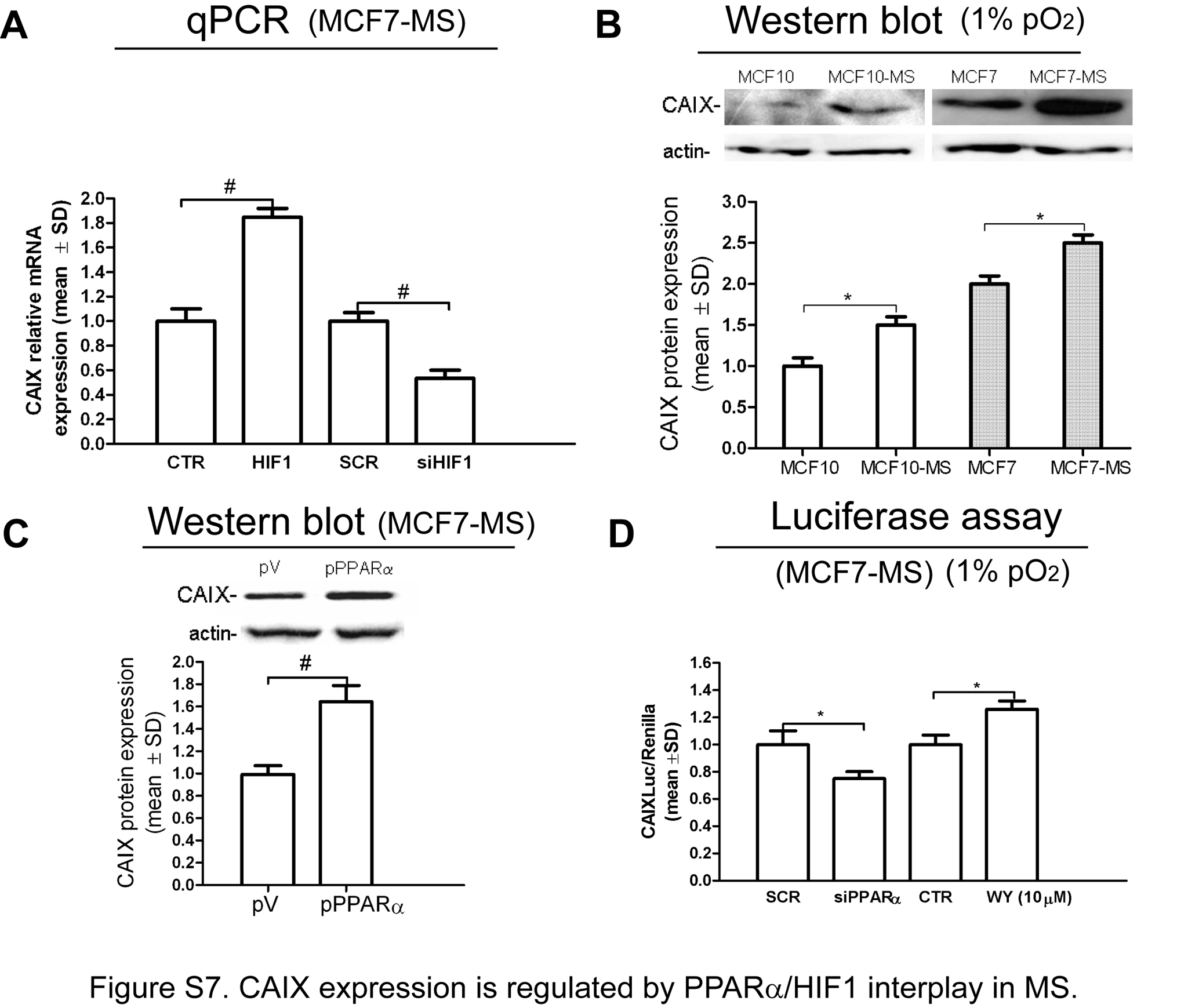

Supplement: Figure S7 — CAIX expression is regulated by PPARα/HIF1 interplay in MS. (A) CAIX mRNA qPCR analysis in HIF1 vector (24 h) or SCR/siHIF1 (72 h)-transfected MCF7-MS. (B) WB analysis of CAIX protein expression in hypoxia exposed MCF10, MCF10-MS, MCF7 and MCF7-MS (C), and in pV/pPPARα (24 h)-transfected MCF7-MS (D). CAIXLuc assay SCR/siPPARα (72 h)-transfected and WY (10 µM, 24 h)-exposed hypoxic MCF7-MS. Data are expressed as mean ±S.D., n = 3, *p<0.05, #p<0.01, ANOVA test. (TIF) [file pone.0054968.s007.tif]

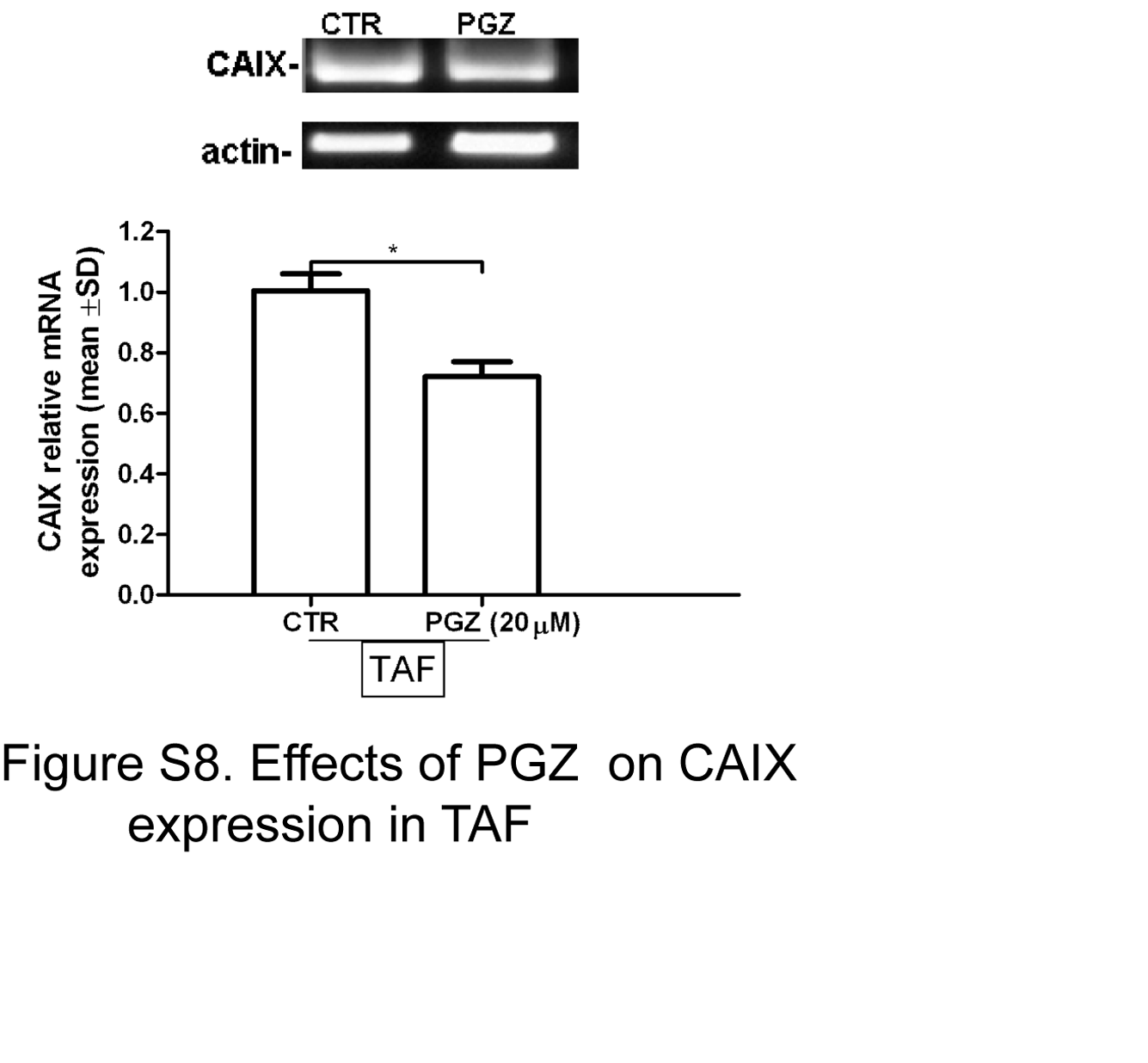

Supplement: Figure S8 — Effects of PGZ on CAIX expression in TAF. CAIX mRNA RT-PCR analysis in PGZ (20 µM)-exposed hypoxic TAF (24 h, samples 21–22). Data are expressed as mean ±S.D., *p<0.05, ANOVA test. (TIF) [file pone.0054968.s008.tif]

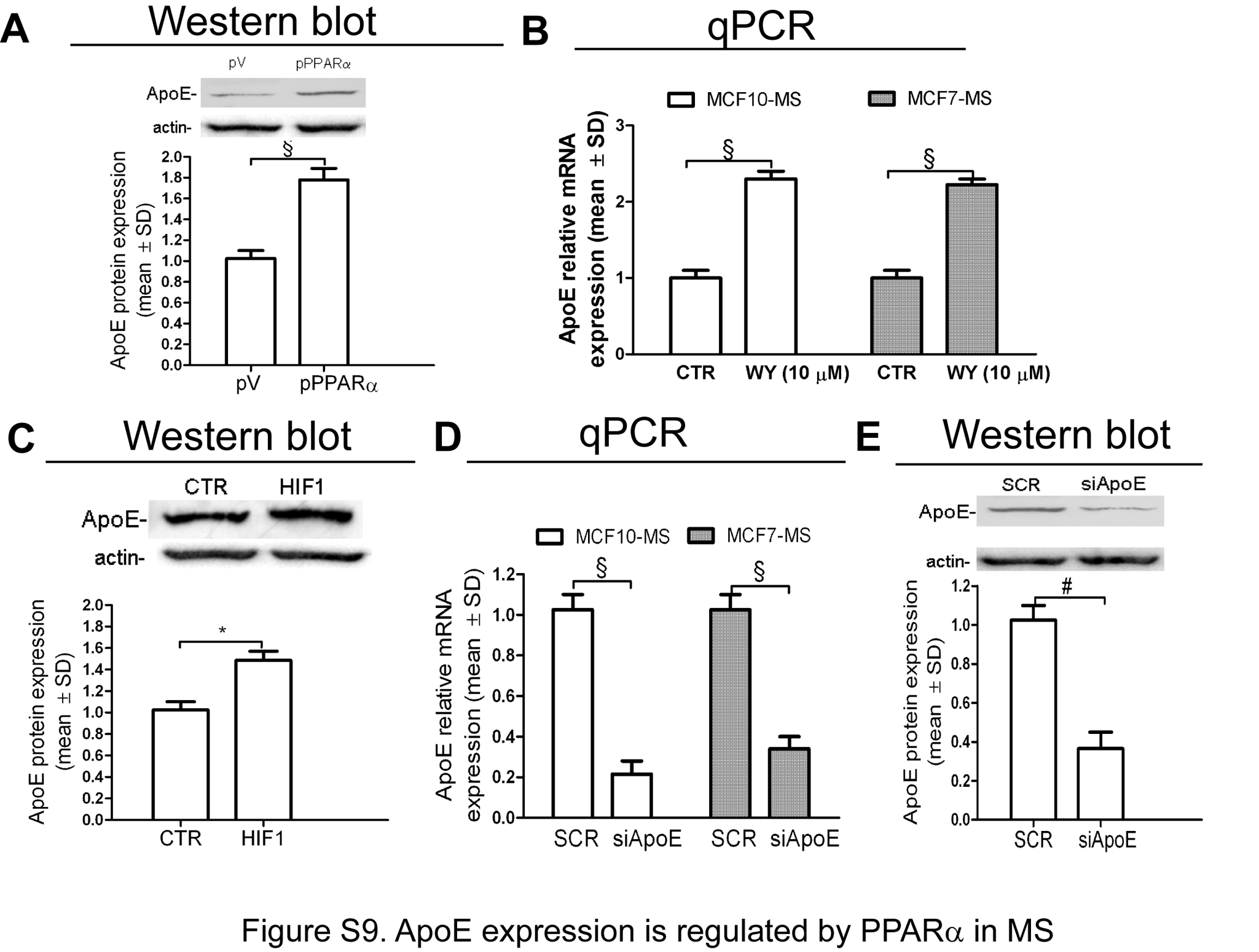

Supplement: Figure S9 — ApoE expression is regulated by PPARα in MS. (A) WB analysis of ApoE protein expression in pV/pPPARα (24 h)-transfected MCF7-MS. (B) ApoE mRNA qPCR analysis in WY (10 µM, 24 h)-exposed MCF10-MS and MCF7-MS. (C) WB analysis of ApoE protein expression in HIF1 vector (48 h)-transfected MCF7-MS. (D) ApoE mRNA qPCR analysis in SCR/siApoE (48 h)-transfected MCF10-MS and MCF7-MS. (E) WB analysis of ApoE protein expression in SCR/siApoE (48 h)-transfected MCF7-MS. Data are expressed as mean ±S.D., n = 3, *p<0.05, # p<0.01, § p<0.005, ANOVA test. (TIF) [file pone.0054968.s009.tif]

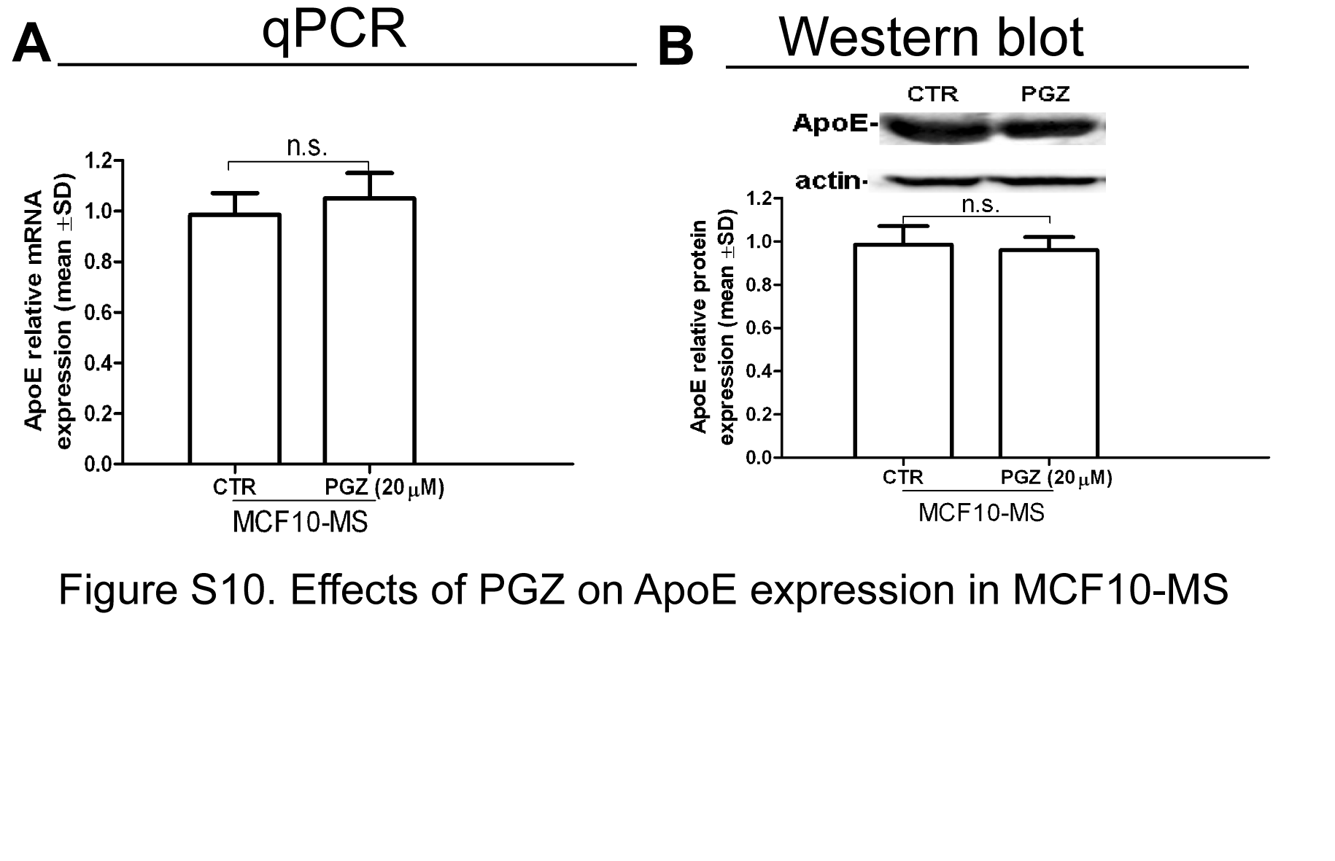

Supplement: Figure S10 — Effects of PGZ on ApoE expression in MCF10-MS. (A) ApoE mRNA qPCR analysis and (B) WB analysis of ApoE protein in PGZ (20 µM, 24 h)-exposed MCF10-MS. Data are expressed as mean ±S.D., n = 3, n.s.: not significant. (TIF) [file pone.0054968.s010.tif]
