# Supplementary material for: Peroxisome Proliferator Activated Receptor-α/Hypoxia Inducible Factor-1α Interplay Sustains Carbonic Anhydrase IX and Apoliprotein E Expression in Breast Cancer Stem Cells
Source: PLoS One. 2013 Jan 25;8(1):e54968. doi: 10.1371/journal.pone.0054968 (PMC3556000; doi:10.1371/journal.pone.0054968)
Supplement: Table S1 — Clinical–pathological parameters of 22 breast carcinomas used for T-MS, N-MS and fibroblasts isolation. List of samples used with clinical and pathological parameters. Abbreviations: pT, tumor size; pN, nodal involvement; G, grade; NG, nuclear grade; ER, estrogen receptor; HER-2, ERbB2 kinase receptor; EGFR, epidermal growth factor receptor. (DOC) [file pone.0054968.s011.doc]

| **Sample** | **Age(yr)** | **pT** | **pN** | **G** | **NG** | **ER** | **HER-2** | **EGFR** |
| --- | --- | --- | --- | --- | --- | --- | --- | --- |
| 1 | 35 | T1 | N2 | G3 | NG3 | 100 | 1 | 0 |
| 2 | 63 | T3 | N1 | G1 | NG1 | 100 | 1 | 0 |
| 3 | 79 | T3 | N1 | G2 | NG2 | 100 | 0 | 0 |
| 4 | 60 | T1 | N0 | G2 | NG2 | 93 | 1 | 0 |
| 5 | 52 | T1 | N0 | G3 | NG3 | 100 | 0 | 0 |
| 6 | 42 | T1 | N0 | G2 | NG2 | 100 | 1 | 0 |
| 7 | 82 | T1 | N0 | G2 | NG3 | 100 | 0 | 0 |
| 8 | 41 | T1 | N0 | G2 | NG3 | 100 | 1 | 0 |
| 9 | 68 | T1 | N0 | G1 | NG1 | 100 | 0 | 0 |
| 10 | 86 | T1 | N1 | G3 | NG3 | 0 | 3 | 0 |
| 11 | 60 | T2 | N3 | G3 | NG3 | 0 | 0 | 2 |
| 12 | 62 | T2 | N0 | G3 | NG3 | 100 | 1 | 0 |
| 13 | 78 | T1 | N1 | G2 | NG3 | 100 | 0 | 0 |
| 14 | 88 | T2 | N1 | G1 | NG2 | 100 | 0 | 0 |
| 15 | 45 | T2 | N3 | G3 | NG3 | 0 | 3 | 2 |
| 16 | 62 | T1 | N0 | G3 | NG3 | 100 | 1 | 0 |
| 17 | 77 | T1 | N0 | G1 | NG2 | 98 | 1 | 0 |
| 18 | 58 | T1 | N3 | G2 | NG3 | 100 | 2 | 0 |
| 19 | 47 | T1 | N0 | G2 | NG3 | 100 | 2 | 0 |
| 20 | 76 | T2 | N0 | G3 | NG3 | 0 | 0 | 2 |
| 21 | 72 | T1 | N0 | G1 | NG2 | 100 | 0 | 0 |
| 22 | 58 | T1 | N1 | G2 | NG2 | 100 | 2 | 0 |

**Table S1. Clinical–pathological parameters of 22 breast carcinomas used for T-MS, N-MS and fibroblasts isolation.**

List of samples used with clinical and pathological parameters. Abbreviations: pT, tumor size; pN, nodal involvement; G, grade; NG, nuclear grade;ER, estrogen receptor; HER-2, ERbB2 kinase receptor; EGFR, epidermal growth factor receptor.
