# Supplementary material for: Peroxisome Proliferator Activated Receptor-α/Hypoxia Inducible Factor-1α Interplay Sustains Carbonic Anhydrase IX and Apoliprotein E Expression in Breast Cancer Stem Cells
Source: PLoS One. 2013 Jan 25;8(1):e54968. doi: 10.1371/journal.pone.0054968 (PMC3556000; doi:10.1371/journal.pone.0054968)
Supplement: Table S2 — List of probes used in qPCR analysis. (DOC) [file pone.0054968.s012.doc]

| **Gene** | **Dye** | **Probe code** |
| --- | --- | --- |
| GUS | VIC | Hs00939627_m1 |
| IL6 | FAM | Hs00985639_m1 |
| PPARα | FAM | Hs00947539_m1 |
| PPARγ | FAM | Hs01115513_m1 |
| Jagged1 | FAM | Hs01070032_m1 |
| ERα | FAM | Hs 00174860_m1 |
| SLUG | FAM | Hs00195591_m1 |
| KRT18 | FAM | Hs00174103_m1 |
| TNFα | FAM | Hs00174128_m1 |
| miR17-5p | FAM | 002308 |
| miR130b | FAM | 000456 |
| RNU6B | FAM | 001093 |
| TNFR1 | FAM | Hs01042323_m1 |
| ApoE | FAM | X0104B64CC (IDT) |
| DDX6 | FAM | 88629624 (IDT) |

**Table S2. List of probes used in qPCR analysis.**

.
