# Supplementary material for: Peroxisome Proliferator Activated Receptor-α/Hypoxia Inducible Factor-1α Interplay Sustains Carbonic Anhydrase IX and Apoliprotein E Expression in Breast Cancer Stem Cells
Source: PLoS One. 2013 Jan 25;8(1):e54968. doi: 10.1371/journal.pone.0054968 (PMC3556000; doi:10.1371/journal.pone.0054968)
Supplement: Table S3 — List of primers used in RT-PCR analysis. (DOC) [file pone.0054968.s013.doc]

| **Primer** | **T(°C)ann.** | **bp amp.** | **Forward 5’-3’** | **Reverse 5’-3’** |
| --- | --- | --- | --- | --- |
| actin | 60 | 192 | GGCATCGTGATGGACTCCG | GCTGGAAGGTGGACAGCGA |
| Notch3 | 62 | 93 | AAGGACGTGGCCTCTGGT | TCAGGCTCTCACCCTTGG |
| Jagged1 | 62 | 120 | TCGCTGTATCTGTCCACCTG | AGTCACTGGCACGGTTGTAG |
| IL8 | 60 | 500 | GGCACAGTGGAACAAGGACT | GCTTTCTGATGGAAGAGAGC |
| CAIX | 61 | 600 | CAGGGACAAAGAAGGGGATGAC | TTGGAAGTAGCGGCTGAAGTCA |
| IL6 | 57 | 360 | GAGAAAGGAGACATGTAACAAGAGT | GCGCAGAATGAGATGAGTTGT |
| RXRα | 62 | 421 | CTCTCAGGTTGAACTCACCT | ATCTCTGACAGCCTGTCTCG |
| RXRβ | 62 | 92 | CTCTGGATGATCAGGTCATAT | GCCATCTCGAACATCAATGGA |
| RXRγ | 58 | 200 | GGGAAGCTGTGCAAGAAGAAA | TGGTAGCACATTCTGCCTCAC |
| PPARβ | 60 | 430 | GTCTCGATGTCGTGGATCAC | AACTGCAGATGGGCTGTGAC |
| TNFα | 60 | 360 | GGCCCAGGCAGTCAGATCAT | GGGGCTCTTGATGGCAGAGA |
| HIF1α | 60 | 615 | GGTGGATATGTCTGGGTTGAAAC | TGGGACTATTAGGCTCAGGTGAA |

**Table S3. List of primers used in RT-PCR analysis.**
